# Supplementary material for: Epithelial colonization by gut dendritic cells promotes their functional diversification
Source: Immunity. 2022 Jan 11;55(1):129–144.e8. doi: 10.1016/j.immuni.2021.11.008 (PMC8751639; doi:10.1016/j.immuni.2021.11.008)
Supplement: Document S1. Figures S1–S6 [file mmc1.pdf]

Immunity, Volume *55*

Supplemental information

Epithelial colonization by gut dendritic cells promotes their functional diversification

Claudia A. Rivera, Violaine Randrian, Wilfrid Richer, Yohan Gerber-Ferder, Maria-Graciela Delgado, Aleksandra S. Chikina, Annika Frede, Chiara Sorini, Mathieu Maurin, Hana Kammoun-Chaari, Sara M. Parigi, Christel Goudot, Mar Cabeza-Cabrerizo, Sylvain Baulande, Sonia Lameiras, Pierre Guermonprez, Caetano Reis e Sousa, Marc Lecuit, Hélène D. Moreau, Julie Helft, Danijela Matic Vignjevic, Eduardo J. Villablanca, and Ana-Maria Lennon-Duménil

Figure S1

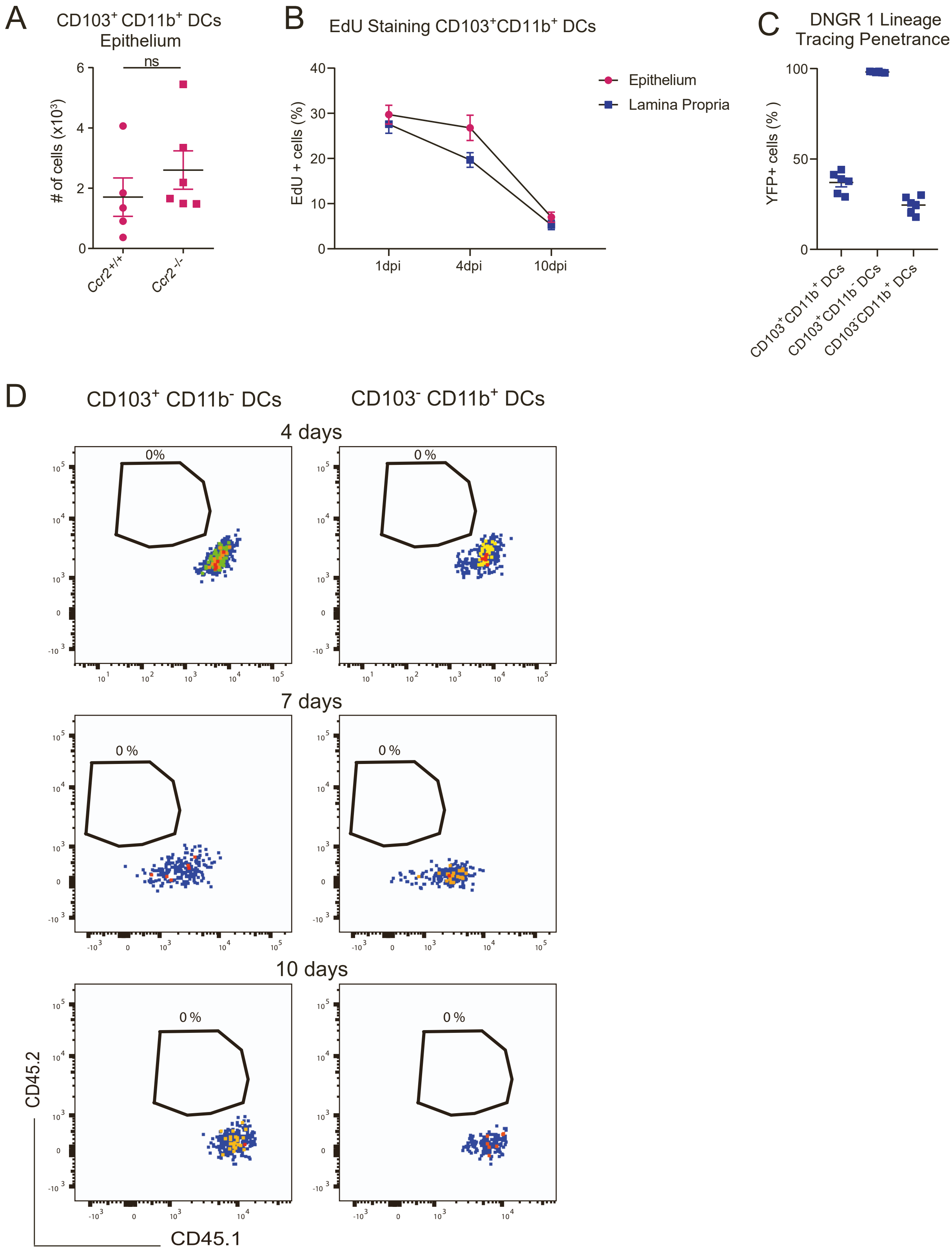

Figure S1 (Related to Figure 3). Analysis of intraepithelial cDC ontogeny. (**A**) Plots of number of $CD103^{+}CD11b^{+}$ in Live $CD45^{+}$ $CD11c^{+}$ $MHCII^{+}$ $CD64^{-}$ cells from the epithelium of the whole small intestine in *Ccr2*$^{+/+}$ and *Ccr2*$^{-/-}$ mice. Data are pooled from two independent experiments. Data were compared using t-test. Each symbol represents one mouse. (**B**) Intraepithelial and LP $CD103^{+}CD11b^{+}$ DCs lifespan analysis after a single EdU I.P. injection in C57BL/6J WT mice. Plot represent the percentage of EdU^{+} cells after 1,4, and 10 days post injection. Date are pooled from 3 independent experiments with n = 2-3 mice per experiment. (**C**) Extent of DNGR 1 fate mapping measured as percentage of YFP^{+} cells in $CD103^{+}CD11b^{+}$, $CD103^{+}CD11b^{-}$ and $CD103^{-}$ $CD11b^{+}$cells (Live $CD45^{+}$ $CD11c^{+}$ $MHCII^{+}$ $CD64^{-}$) from the lamina propria of the whole small intestine of *Clec9a*$^{+/cre}$Rosa$^{+/EYFP}$ mice. (**D**) Representative flow cytometry dot plots of CD45.2 preDC transfer experiments showing differentiation dynamics in the small intestine epithelium of syngeneic CD45.1 mice. Differentiation of preDCs into $CD103^{+}CD11b^{-}$ and $CD103^{-}CD11b^{+}$ DCs was followed after 4, 7, and 10 days post-transfer.

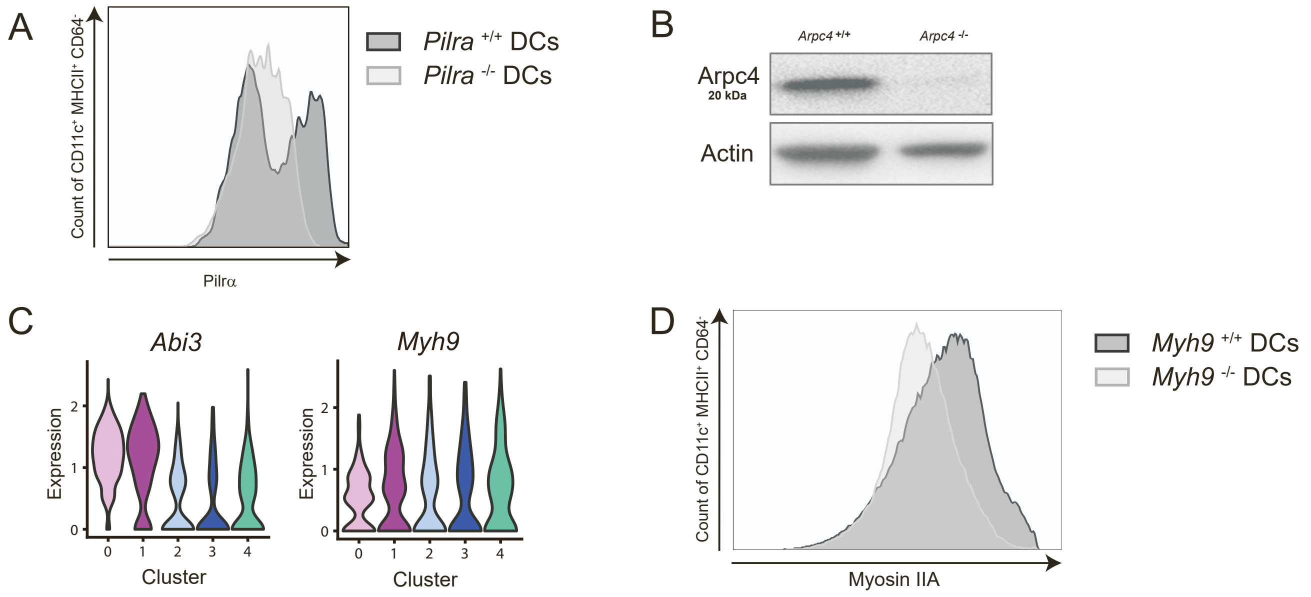

Figure S2 (Related to Figure 4). Transmigration-related molecules characterization. (**A**) Example of Pilralpha surface staining in CD11c$^+$MHCII$^+$CD64$^-$ cells from the small intestine of *Pilra*$^{+/+}$ and *Pilra*$^{-/-}$ mice. (**B**) Western blot of the Arp2/3 complex molecule Arpc4 show that *Arpc4*$^{flox/flox}$ x *Itgax*$^{Cre+}$ (*Arpc4*$^{-/-}$) cells do not express the Arpc4 protein in BM derived dendritic cells compared to the *Arpc4*$^{flox/flox}$ x *Itgax*$^{Cre-}$ (*Arpc4*$^{+/+}$) counterpart. (**C**) Violin plots representing expression of *Abi3* and *Myh9* (MyoIIA) among clusters. (**D**) Example of intracellular Myosin IIA staining in CD11c$^+$MHCII$^+$CD64$^-$ cells from the small intestine of *Myh9*$^{+/+}$ (*Myh9*$^{flox/flox}$ x *Itgax*$^{Cre-}$) and *Myh9*$^{-/-}$ (*Myh9*$^{flox/flox}$ x *Itgax*$^{Cre+}$) mice.

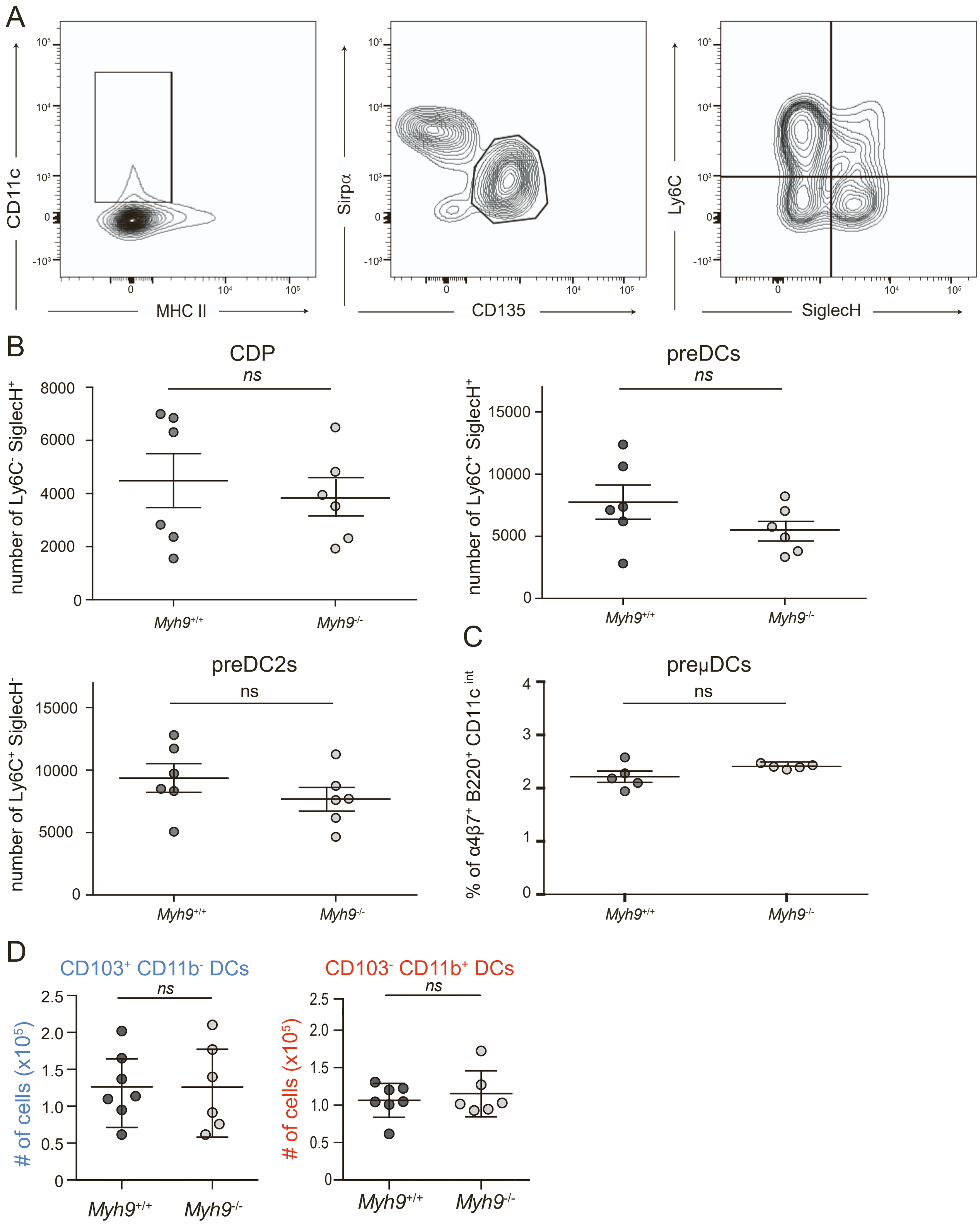

Figure S3 (Related to Figure 4). *Myh9*$^{-/-}$ mice characterization. (**A**) Gating strategy used to characterize common DC progenitors (CDP) and preDCs from Live Lineage (CD3, CD19, NKP46, Ter119, Ly6G, B220) negative cells in bone marrow of *Myh9*$^{+/+}$ (*Myh9*$^{flox/flox}$ x *Itgax*$^{Cre-}$) and *Myh9*$^{-/-}$ (*Myh9*$^{flox/flox}$ x *Itgax*$^{Cre+}$) mice. (**B**) Plot of number of Ly6C^{-}SiglecH^{+} in Live Lineage^{-} cells (CDP) (left top panel), Ly6C^{+}SiglecH^{+} in Live Lineage^{-} cells (preDCs) and Ly6C^{+}SiglecH^{-} in Live Lineage^{-} cells (preDC2s), in bone marrow of *Myh9*$^{+/+}$ and *Myh9*$^{-/-}$ mice. Data are pooled from two independent experiments and compared using Paired t-test and Welch's test. Each symbol represents one mouse. (**C**) Plot of percentage of α4β7^{+}B220^{+}CD11c^{int} in Live Lineage (CD3, CD19, NKP46, Ter119, Ly6G) negative cells (preμDCs) in bone marrow of *Myh9*$^{+/+}$ and *Myh9*$^{-/-}$ mice. Data are pooled from two independent experiments and compared using Paired t test. Each symbol represents one mouse. (**D**) Plots of number of CD103^{+}CD11b^{-} (cDC1s) and CD103^{-}CD11b^{+} (classical cDC2s) in Live CD45^{+} CD11c^{+} MHCII^{+} CD64^{-} cells from the lamina propria of the whole small intestine in *Myh9*$^{+/+}$ and *Myh9*$^{-/-}$ mice. Data are pooled from three independent experiments. Data were compared using t-test. Each symbol represents one mouse.

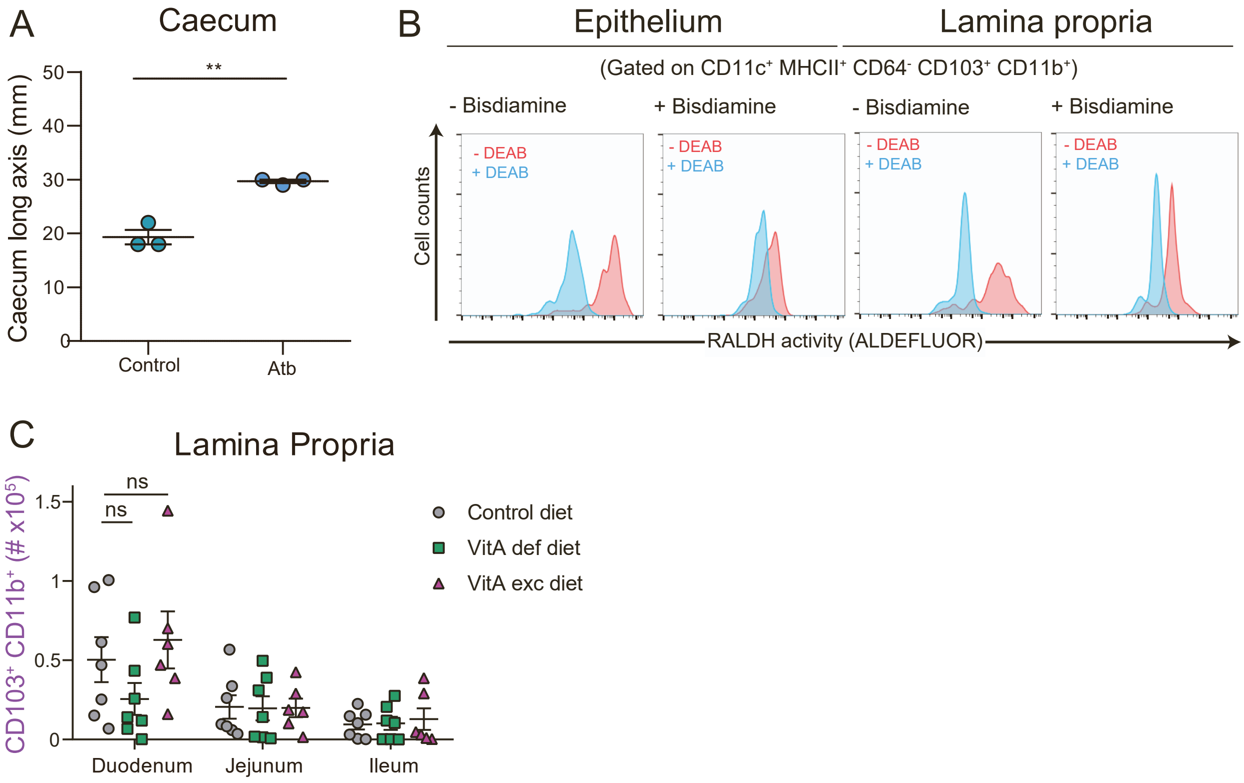

Figure S4 (Related to Figure 5). Environmental factors modulation of cDC2 transmigration. (**A**) Caecum size of adult SPF C57BL/6J mice gavage with PBS (control) or a cocktail of antibiotics. Data were compared using t-test. (**B**) Plots represent an example of epithelium (left) and lamina propria (right) from the small intestine of an adult SPF C57BL/6J mice treated with Bisdiamine or olive oil only. RALDH inhibitor (DEAB) provided with ALDEFLUOR kit was used as a negative control to set up gates for each sample. (**C**) Flow cytometry analysis of $CD103^{+}CD11b^{+}$ DCs numbers from the small intestinal lamina propria analyzed in duodenum, jejunum and ileum of SPF C57BL/6J mice fed with Vitamin A deficient, excess or control diet for 3 months. Data are pooled from 2 independent experiments and compared using two-way ANOVA. Each symbol represents one mouse.

Figure S5

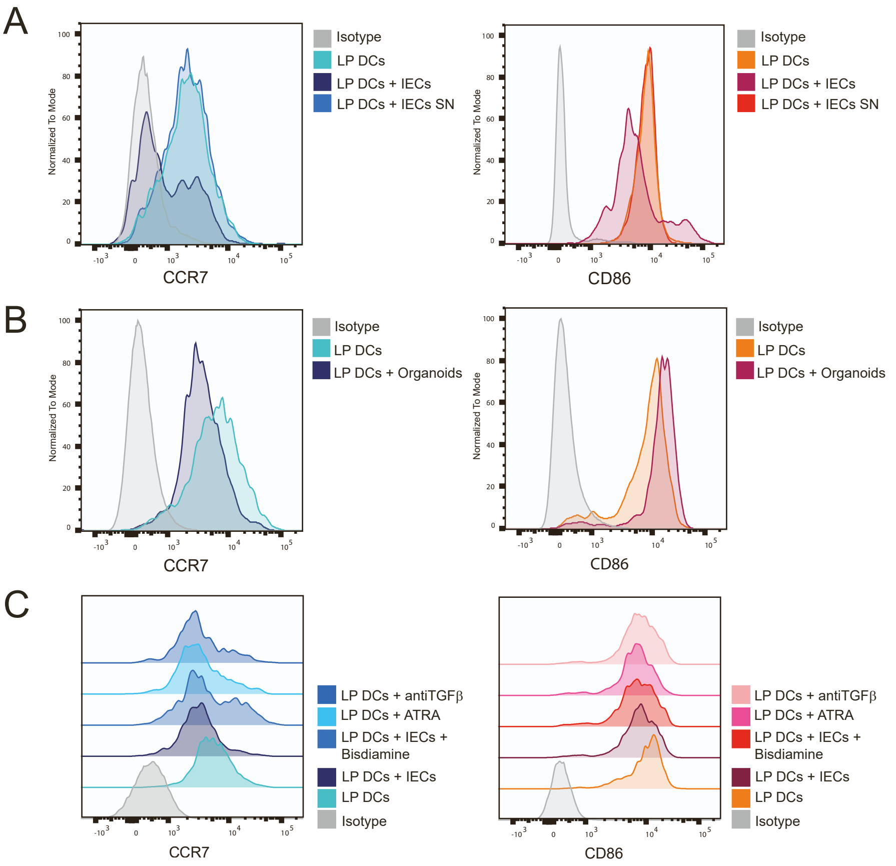

Figure S5 (Related to Figure 6). CD103^{+}CD11b^{+} cDC2s epithelial imprinting. (**A**) Histogram plots representing an example of CCR7 and CD86 expression after co-culture of LP CD103^{+}CD11b^{+} DCs with small intestine epithelial cells or supernatant of small intestine epithelial cells. (**B**) Histogram plots representing an example of CCR7 and CD86 expression after co-culture of LP CD103^{+}CD11b^{+} DCs with small intestinal duodenal organoids. (**C**) Histogram plots showing examples of CCR7 and CD86 expression after incubation of LP CD103^{+}CD11b^{+} DCs with small intestine epithelial cells in the presence or absence of ATRA, Bisdiamine and anti TGFβ treatments.

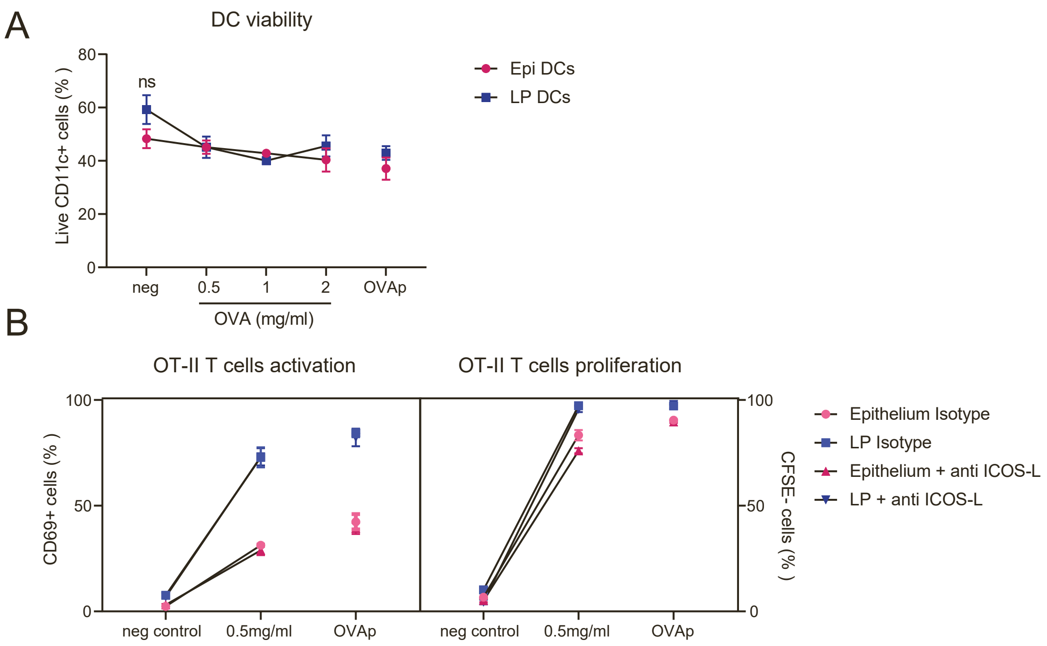

Figure S6 (Related to Figure 7). Blocking ICOS-L does not increase T cell activation or proliferation induced by intraepithelial cDC2s. (**A**) Percentage of Live CD11c^{+} cells in antigen presentation assays after overnight co-culture of CD103^{+}CD11b^{+} DCs with OT-II T cells. Data are pooled from 2 independent experiments. (**B**) OT-II antigen presentation assay performed with sorted CD103^{+}CD11b^{+} DCs from both small intestine lamina propria and epithelium from C57BL/6J mice, showing percentage of activation (Left panel) and proliferation (Right panel) after incubation of DCs with anti ICOS-L and isotype antibodies at the same concentration (50 ug/ml). Data are pooled from 2 independent experiments.
