# Supplementary material for: Epithelial colonization by gut dendritic cells promotes their functional diversification
Source: Immunity. 2022 Jan 11;55(1):129–144.e8. doi: 10.1016/j.immuni.2021.11.008 (PMC8751639; doi:10.1016/j.immuni.2021.11.008)
Supplement: Document S2. Article plus supplemental information [file mmc3.pdf]

Immunity

Article

# Epithelial colonization by gut dendritic cells promotes their functional diversification

## Graphical abstract

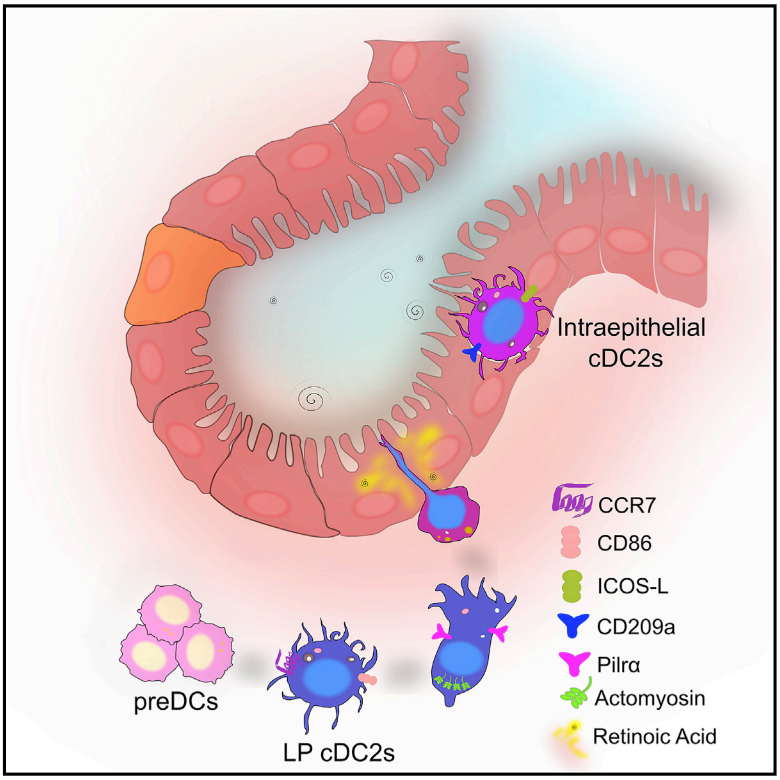


## Highlights

- Epithelial colonization by gut cDC2s leads to their transcriptional reprograming
- Unlike lamina propria cDC2s, intraepithelial cDC2s show an immature-like phenotype
- Intraepithelial cDC2s trigger T cell hyporesponsiveness
- The phenotype of intraepithelial cDC2s is imprinted by both retinoic acid and mucus

## Authors

Claudia A. Rivera, Violaine Randrian, Wilfrid Richer, ..., Danijela Matic Vignjevic, Eduardo J. Villablanca, Ana-Maria Lennon-Duménil

## Correspondence

amlennon@curie.fr

## In brief

Gut cDC2s have been described to migrate into the epithelium, but whether this event modifies their phenotype is unknown. Rivera et al. show that upon epithelium colonization, cDC2s adopt an immature-like phenotype, revealing the existence of subtissular niches able to shape cDCs' fate and function.

Rivera et al., 2022, Immunity *55*, 129–144
January 11, 2022 © 2021 The Authors. Published by Elsevier Inc.
https://doi.org/10.1016/j.immuni.2021.11.008

CellPress

Immunity

CellPress OPEN ACCESS

Article

# Epithelial colonization by gut dendritic cells promotes their functional diversification

Claudia A. Rivera,[1] Violaine Randrian,[1] Wilfrid Richer,[1] Yohan Gerber-Ferder,[1] Maria-Graciela Delgado,[1] Aleksandra S. Chikina,[1,2] Annika Frede,[3,4] Chiara Sorini,[3,4] Mathieu Maurin,[1] Hana Kammoun-Chaari,[5] Sara M. Parigi,[3,4] Christel Goudot,[1] Mar Cabeza-Cabrerizo,[6] Sylvain Baulande,[7] Sonia Lameiras,[7] Pierre Guermonprez,[8] Caetano Reis e Sousa,[6] Marc Lecuit,[5,9] Hélène D. Moreau,[1] Julie Helft,[1] Danijela Matic Vignjevic,[2] Eduardo J. Villablanca,[3,4] and Ana-Maria Lennon-Duménil[1,10,*]

[1]Institut Curie, INSERM U932, PSL Research University, 75005 Paris, France
[2]Institut Curie, CNRS UMR 144, PSL Research University, 75005 Paris, France
[3]Immunology and Allergy division, Department of Medicine, Solna, Karolinska Institutet and University Hospital, 17176 Stockholm, Sweden
[4]Center of Molecular Medicine, 17176 Stockholm, Sweden
[5]Biology of Infection Unit, Institut Pasteur, INSERM U1117, 75015 Paris, France
[6]Immunobiology Laboratory, The Francis Crick Institute, London NW1 1AT, UK
[7]ICGex Next-Generation Sequencing Platform, Institut Curie, PSL Research University, 75005 Paris, France
[8]Université de Paris, Centre for Inflammation Research, CNRS ERL8252, INSERM1149, Paris, France
[9]Université de Paris, Necker-Enfants Malades University Hospital, Department of Infectious Diseases and Tropical Medicine, APHP, Institut Imagine, Paris, France
[10]Lead contact
*Correspondence: amlennon@curie.fr
https://doi.org/10.1016/j.immuni.2021.11.008

## SUMMARY

Dendritic cells (DCs) patrol tissues and transport antigens to lymph nodes to initiate adaptive immune responses. Within tissues, DCs constitute a complex cell population composed of distinct subsets that can exhibit different activation states and functions. How tissue-specific cues orchestrate DC diversification remains elusive. Here, we show that the small intestine included two pools of cDC2s originating from common pre-DC precursors: (1) lamina propria (LP) CD103$^+$CD11b$^+$ cDC2s that were mature-like proinflammatory cells and (2) intraepithelial cDC2s that exhibited an immature-like phenotype as well as tolerogenic properties. These phenotypes resulted from the action of food-derived retinoic acid (ATRA), which enhanced actomyosin contractility and promoted LP cDC2 transmigration into the epithelium. There, cDC2s were imprinted by environmental cues, including ATRA itself and the mucus component Muc2. Hence, by reaching distinct subtissular niches, DCs can exist as immature and mature cells within the same tissue, revealing an additional mechanism of DC functional diversification.

## INTRODUCTION

Conventional dendritic cells (cDCs) were initially described for their capacity to patrol peripheral tissues and transport the antigens collected to lymph nodes for presentation to T lymphocytes. This process constitutes the first step of adaptive immune responses. The cDCs that reside in most peripheral tissues are in the so-called “immature stage”: they exhibit a high antigen internalization capacity and express low amounts of costimulatory molecules, proinflammatory cytokines, and the chemokine receptor CCR7 (Cabeza-cabrerizo et al., 2021). Upon detecting danger-associated antigens, these cDCs enter into a maturation program that downregulates antigen internalization and enhances surface expression of CCR7, resulting in cDC migration to lymph nodes for encounter with their intended T cells. cDC maturation also promotes the expression of costimulatory molecules and proinflammatory cytokines that endow them with the capacity to activate these lymphocytes.

This picture was later deepened as it has been described that, in both mouse and human, cDCs form a highly heterogeneous cell population within peripheral tissues, with the existence of different DC subtypes exhibiting different transcriptional programs, activation states, and functions. They can be divided into two major categories: cDC1s (CD11c$^{hi}$MHCII$^{hi}$CD103$^+$ CD11b$^-$) and cDC2s (CD11c$^{hi}$MHCII$^{hi}$CD103$^-$CD11b$^+$), differentiating from pre-cDC1 and pre-cDC2 precursors, respectively (Schlitzer et al., 2015). The case of the small intestine is particularly appealing as, in homeostasis, lamina propria (LP) cDCs also include a cDC2 population that expresses both CD103 and CD11b markers and is more abundant than classical cDC2s (Bogunovic et al., 2009; Persson et al., 2013).

Gut cDC differentiation is dictated by cell-intrinsic properties as well as environmental cues (Heidkamp et al., 2016; Klebanoff


This is an open access article under the CC BY license (http://creativecommons.org/licenses/by/4.0/).

et al., 2013). The ontogeny of both pools of LP cDC2s is driven by the transcription factor IRF4 and requires the expression of Notch2 (Lewis et al., 2011; Schlitzer et al., 2013). However, unlike their classical cDC2 counterpart, $CD103^+CD11b^+$ cDC2s also need local production of vitamin A-derived all-*trans*-retinoic acid (ATRA), their number being 50% reduced in the small intestine of mice that do not synthesize this metabolite (Klebanoff et al., 2013). ATRA is produced by different cell types in the small intestine, including $CD103^+CD11b^+$ cDC2s themselves and epithelial cells, following a proximal-distal gradient in which higher ATRA concentrations are found in the duodenum (Villablanca et al., 2011). In addition to its role in the homeostasis of $CD103^+CD11b^+$ cDC2s, ATRA has been shown to regulate gut immunity by promoting gut-specific homing of T lymphocytes and facilitating the generation of $FoxP3^+$ regulatory T (Treg) cells (Esterházy et al., 2016; Hall et al., 2011). Whether these different effects of ATRA are linked or not is unclear, as well as its precise mechanism of action.

It has been shown by intravital two-photon imaging that $CD103^+CD11b^+$ cDC2s can migrate from the LP into the epithelium of the small intestine (Farache et al., 2013). This migration event can take place in homeostasis and is further enhanced upon oral challenge with *Salmonella*. Once in the epithelium, cDC2s can capture luminal *Salmonella*. How this migratory event impacts the identity and function of $CD103^+CD11b^+$ cDC2s has not been addressed. More generally, how the localization of cDCs to distinct subtissular niches might influence their exposure to local environmental cues, thus shaping their differentiation and fate, remains largely unknown.

Here, we investigated how the colonization of the small intestine epithelium by $CD103^+CD11b^+$ cDC2s contributes to their functional diversification. Using single-cell RNA sequencing analysis, we show that LP and intraepithelial $CD103^+CD11b^+$ cDC2s display distinct transcriptomic profiles and different functions: while LP $CD103^+CD11b^+$ cDC2s display a mature proinflammatory phenotype and, accordingly, promote T cell activation, intraepithelial $CD103^+CD11b^+$ cDC2s are immature and rather trigger T cell anergy. We further identify ATRA as the master regulator of this process: ATRA promotes myosin IIA-dependent contractility, facilitating cDC2 transmigration into the epithelium where they are exposed to local cues that imprint their immature anti-inflammatory phenotype. These results show that the localization of DCs to different subtissular niches controls their functional diversification and allows them to co-exist in different maturation states within a given tissue.

## RESULTS

### Lamina propria and intraepithelial cDC2s exhibit distinct transcriptional profiles

Intravital live-imaging has shown that, in the small intestine, a fraction of LP cDCs can colonize the epithelium in homeostasis, leading to the formation of a pool of intraepithelial cDCs (Farache et al., 2013). These cells mainly belong to the $CD103^+CD11b^+$ cDC2 subtype, as confirmed by flow cytometry analyses performed upon epithelium-LP separation (Figure 1A) and whole-mount immunostaining of gut sections (Figure 1B). To investigate whether epithelial colonization impacts the activation state and transcriptional programing of DCs, we compared the gene expression profiles of sorted intraepithelial $CD103^+CD11b^+$ cDC2s with those of their LP counterparts. As the degree of heterogeneity of these gut cDC2 populations was unknown, we used a droplet-based method that enables 3′ mRNA counting for single-cell RNA sequencing (10x genomics; Zheng and Tian, 2017). Our data set collected a total of 1,263 cells, including 674 cells from the LP DC sample and 589 cells from the epithelial one. The t-distributed stochastic neighbor embedding (tSNE) analysis showed that the transcriptional profiles of these two samples were unambiguously distinct (Figure 1C). Clustering (see STAR Methods) defined a total of 5 clusters for the two samples (Figure 1D; Table S1): clusters 0 and 1 corresponded almost exclusively to the epithelial DC sample, whereas clusters 2, 3, and 4 were constituted by >95% of cDCs from the LP (Figure 1E). CD11c (*Itgax*), CD103 (*Itgae*), and CD11b (*Itgam*) were expressed similarly in all clusters (Figure 1F). These results suggest that epithelial colonization might transcriptionally shape gut cDC2s.

### Lamina propria cDC2s display a proinflammatory mature-like gene signature

Analysis of the clusters corresponding to LP $CD103^+CD11b^+$ cDC2s (clusters 2–4) showed that cluster 2 was enriched for cytokine signaling genes (Reactome 2016, p value 2,29e-5), including genes from the interleukin-1 (IL-1) pathway (Wiki Pathway 2015, p value 1,24e-3) (Figure 2A). This proinflammatory signature was even more pronounced in LP cluster 3, which further showed elevated expression of genes related to the tumor necrosis factor alpha (TNF$\alpha$) and nuclear factor (NF-$\kappa$B) pathways (Wiki Pathway 2016, p value 3,589e–7) (Figure 2A, proinflammatory genes highlighted in blue, and Figure 2B). This cluster was the only one expressing high amounts of CCR7 and its positive regulator Nr4a3 (Figures 2A and 2C), suggesting that they could migrate to lymph nodes at a steady state. This result is consistent with previous reports showing that the cDCs migrating from the small intestine to lymph nodes mainly belong to the $CD103^+CD11b^+$ cDC2s subtype (Liu et al., 2007; Mazzini et al., 2014); yet, it suggests that this concerns only a fraction of this cell population, at least under homeostatic conditions. Increased expression of CCR7 in LP $CD103^+CD11b^+$ cDC2s was confirmed by flow cytometry using a $Ccr7^{gfp}$ reporter mouse (Nakano et al., 2013) (Figure 2D, left panel). The elevated expression of the DC maturation marker CD83 (Figure 2D, right panel), as well as the increased expression of proinflammatory cytokines TNF$\alpha$ and IL-1$\beta$ by LP $CD103^+CD11b^+$DCs, was also corroborated (Figure 2E). LP cDC2s also showed higher expression of the costimulatory molecules CD86 and CD80 than intraepithelial cDC2s (Figure 2F). Although proinflammatory genes were enriched in cluster 4, they did not reach the high expression found in clusters 2 and 3. This cluster was strongly enriched for cell cycle genes (Kegg 2016, p value 3,275e–6; Reactome 2016, p value 1,467e–25) (Figure 2A, cycling genes in green), in agreement with earlier studies highlighting a subset of cycling cells among differentiated cDCs in the periphery, including the LP (Cabeza-Cabrerizo et al., 2019; Kabashima et al., 2005; Liu et al., 2007). These results show that LP $CD103^+CD11b^+$ cDC2s express proinflammatory markers, high amounts of costimulatory molecules, and the chemokine receptor CCR7, thus harboring a mature-like DC phenotype.

CellPress
OPEN ACCESS

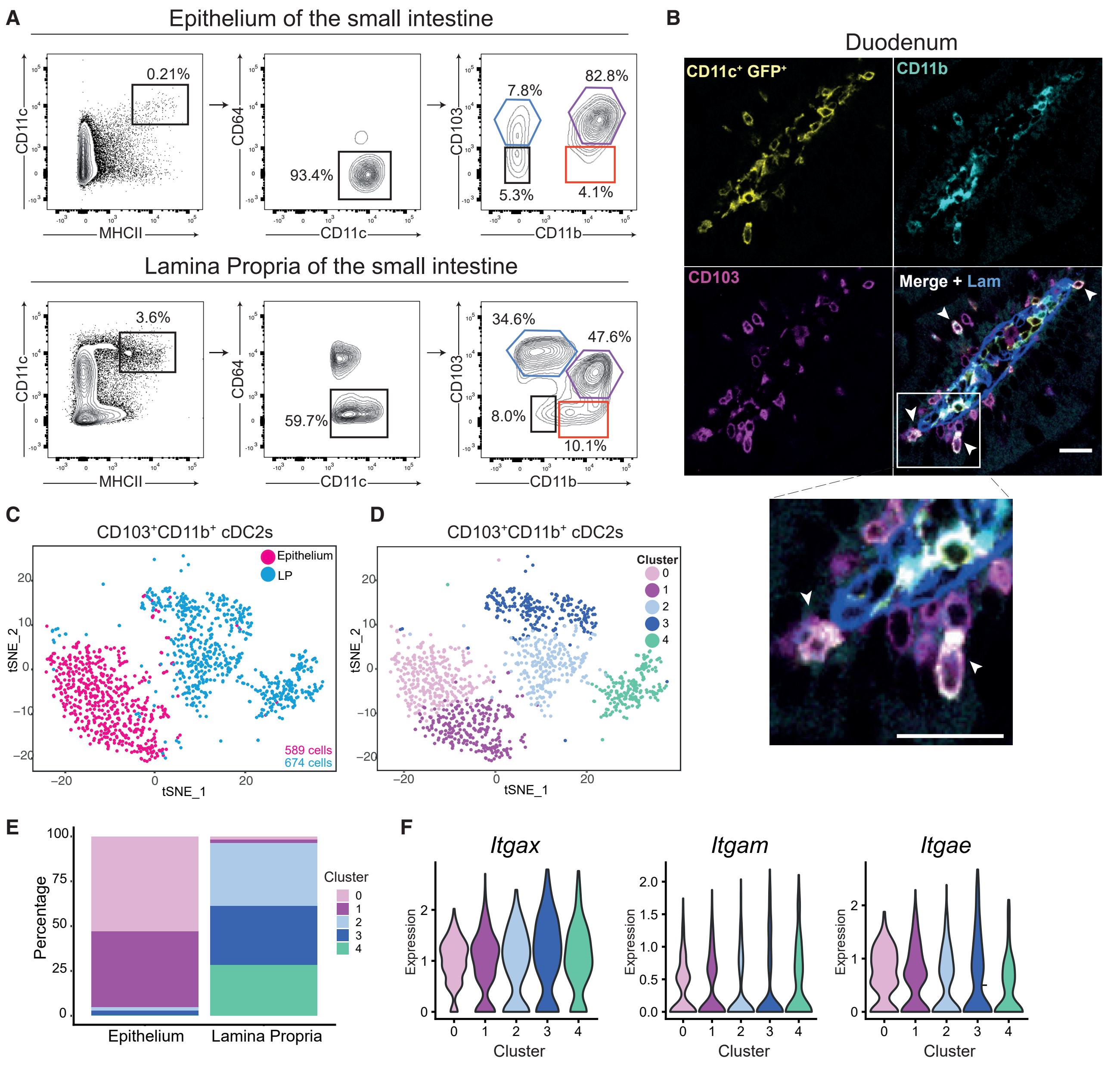


**Figure 1. LP and intraepithelial CD103b$^+$CD11b$^+$ cDC2s have distinct transcriptomic profiles**

(A) Gating strategy used to characterize DCs in the epithelium and lamina propria of the whole small intestine from live CD45$^+$ cells: cDC1s (CD103$^+$CD11b$^-$), cDC2s (CD103$^+$CD11b$^+$ and CD103$^-$CD11b$^+$), and double negative CD103$^-$CD11b$^-$ DCs. Example of C57BL/6J mouse.

(B) Image of fixed slices from the duodenum of *Itgax*: Cre/R26$^{mTmG}$ mice. Sections represent CD11c (yellow), CD11b (cyan), CD103 (magenta), and laminin (blue). Bottom right panel represents merged images. Scale bar, 20 µm. Representative of 3 independent experiments.

(C and D) Purified CD11c$^+$CD103$^+$CD11b$^+$ cDCs were analyzed by single-cell RNA-seq using a drop-seq approach. Colors represent samples identification (C) and unbiased clustering from graph-based clustering with resolution of 1 (D). Each dot represents an individual cell from a single experiment. tSNE analysis of individual cells for total cells (n = 1,263).

(E) Barplot represents the percentage of cells in each cluster found in each sample.

(F) Violin plots showing the expression of *Itgax* (A), *Itgam* (B), and *Itgae* (C) in different clusters obtained by unbiased analysis.

## Intraepithelial cDC2s resemble immature cDCs

In contrast to their LP counterparts, intraepithelial cDC2s (clusters 0 and 1) were not enriched for proinflammatory genes (Figures 2A and 2B). These two clusters displayed similar gene expression profiles, but cluster 0 showed even lower expression of proinflammatory genes than cluster 1. They were both enriched in C-type lectin genes such as *Cd209a* (murine DC-SIGN), antimicrobial peptide genes (*Rnase6*, *Defa24*), and phagolysosome maturation-associated genes (Figures 2A and 2C, genes in orange) (GO Cellular Component 2017b, p value

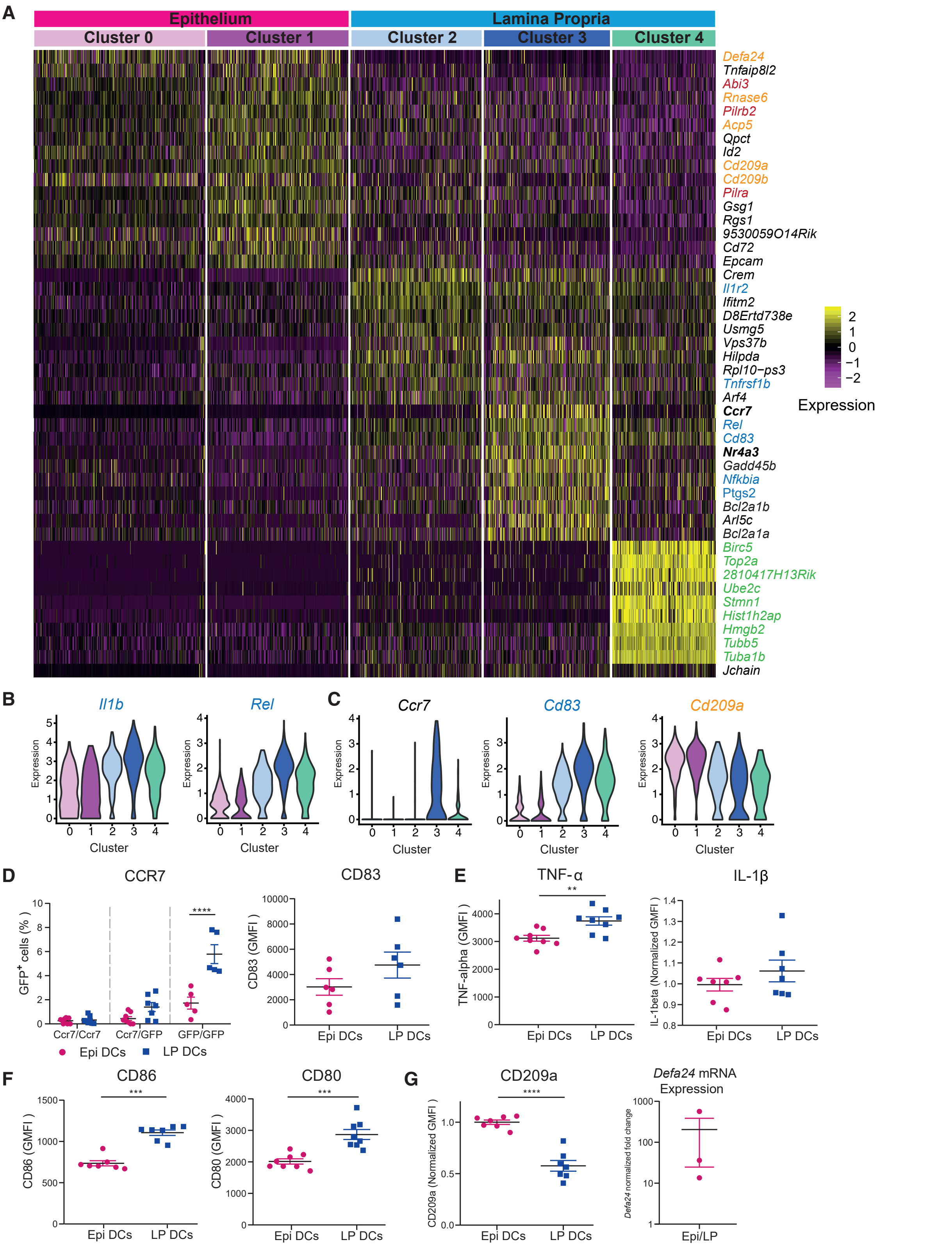


**Figure 2. While LP CD103⁺CD11b⁺ cDC2s express proinflammatory markers, intraepithelial cDC2s display an anti-inflammatory phenotype**
(A) Heatmap of scaled expression (log normalized UMI counts) of the top 20 most differentially expressed genes for each cluster. Antimicrobial genes are highlighted in orange, migration genes in red, inflammatory genes in blue, and cyclin genes in green.

(legend continued on next page)

CellPress
OPEN ACCESS

6,28e-3). None of these genes were highly expressed in LP cDC2s. These data were validated by flow cytometry for CD209a and quantitative reverse transcription PCR for the antimicrobial peptide defensin 24 (Defa24) (Figure 2G). They revealed that epithelial colonization by gut CD103$^+$CD11b$^+$ cDC2s was associated with modifications of their gene expression profile, with decreased expression of proinflammatory genes but enhanced expression of antimicrobial ones, which are typical features of immature DCs.

## Lamina propria and intraepithelial cDC2s have a common origin

To gain more insights into the potential filial relation between LP and intraepithelial CD103$^+$CD11b$^+$ cDC2s, we exploited the divergences observed between both samples to generate single-cell trajectories. For this, we used a pseudotime trajectory algorithm (monocle 2, Qiu et al. 2017), including all the differentially expressed genes observed between the various clusters. This analysis suggested that intraepithelial DC clusters 0 and 1 were distinct from LP DC clusters 2 and 3 (Figure 3A). It further identified cluster 2 as an intermediary cell population located between LP clusters 3 and 4 (both noncycling and cycling) and intraepithelial clusters 0 and 1. Accordingly, cluster 2 exhibited lower expression of inflammatory genes than LP cluster 3 but higher than intraepithelial clusters 0-1. Dynamic analysis using RNA velocity of single cells (La Manno et al., 2018) did not provide any evidence for cluster 4 (cycling cells) giving rise to clusters 0-3 (Figure 3B), suggesting that they might be unrelated. In contrast, this analysis was consistent with cluster 2 indeed corresponding to an intermediate cluster from which cluster 3 and clusters 0-1 could originate. Thus, although nondemonstrative, both monocle and RNA velocity analyses are consistent with a model where cDC2s from cluster 2 differentiate into cells that either remain in the LP and further upregulate proinflammatory genes and (cluster 3) becoming mature-like cDC2s, or migrate into the epithelium and downregulate these genes (clusters 0-1), leading to the emergence of an immature-like intraepithelial cDC2 population (Figure 3B).

Different experimental approaches were undertaken to challenge this model. First, we verified that, as LP cDC2s, intraepithelial cDC2s originated from pre-DCs by assessing whether they relied on the presence of the Flt-3 ligand cytokine, which was indeed confirmed (Figure 3C). Accordingly, we observed that intraepithelial cDC2s do not rely on CCR2, excluding their monocytic origin (Figure S1A) and that they exhibit a similar lifespan to CD103$^+$CD11b$^+$ LP cDC2s (Figure S1B). Next, we used the Clec9a-RosaEYFP mouse for lineage tracing. CLEC9A (also known as DNGR-1) is a membrane C-type lectin receptor expressed early and specifically during cDC development from common DC progenitors (CDPs). Although CLEC9A is lost in differentiated cDC2s, it can be used to trace cDCs, as it is not expressed by other leukocytes (Schraml et al., 2013). Remarkably, analysis of the percentage of YFP-positive CD103$^+$CD11b$^+$ cDC2s in the LP and epithelium of CLEC9A-RosaEYFP mice showed that they were similar (Figure 3D). This was in contrast to LP cDC1s that displayed a higher percentage of YFP$^+$ cells (Figure S1C), as expected from previous reports (Schraml et al., 2013). These results strongly suggest that intraepithelial and LP CD103$^+$CD11b$^+$ cDC2s have a common origin.

To provide direct experimental evidence for this, we purified bone marrow (BM) pre-DCs and adoptively transferred them into syngeneic recipients. Mice used for pre-DC purification were inoculated beforehand with Flt3l-producing tumor cells to increase the size of their pre-DC compartment (Scott et al., 2015). Analysis of recipient animals showed that transferred CD103$^+$CD11b$^+$ cDC2s were detected in the LP as early as 4 days after transfer, with no intraepithelial cDC2 being found at that time (Figure 3E). Intraepithelial cDC2s became, however, detectable 7 days after transfer and, as LP cDC2s, increased up to day 10 (Figure 3F). Of note, no pre-DC-derived intraepithelial cDC1s or CD103$^-$CD11b$^+$ cDC2s were detected at any point after the transfer (Figure S1D). These results suggested that LP and intraepithelial CD103$^+$CD11b$^+$ cDC2s most likely originated from common pre-DCs colonizing first at the LP and then at the epithelium, rather than from distinct precursors. They were in good agreement with RNA-seq trajectories suggesting that pre-DCs arrived from the bone marrow to the small intestine LP (where blood vessels are) to form a transitional pool of CD103$^+$CD11b$^+$ cDC2s (cluster 2), which then either remained in the LP to become proinflammatory mature cDC2s (cluster 3) or moved to the epithelium to generate a pool of immature cDC2s (clusters 0-1) (Figure 3G).

## Colonization of the epithelium by cDC2s relies on PILR$\alpha$ and the actomyosin cytoskeleton

We next zoomed into the genes enriched in intraepithelial cDC2 clusters 0 and 1 to search for candidates involved in transmigration and epithelium colonization. Both clusters were enriched for expression of genes involved in (1) leukocyte transmigration through endothelia (KEGG 2015, p value 0.051), namely paired

(B) Violin plots showing the expression of inflammatory genes: *Il1b* and *Rel* proto-oncogene (NF-κB subunit).

(C) Violin plots showing the expression of *Ccr7*, *CD83*, and *CD209a* among clusters.

(D) Left panel: plot of percentage of GFP positive cells in CD103$^+$CD11b$^+$ cDC2s as reporter of CCR7 expression in *Ccr7*$^{gfp}$ mice lamina propria and epithelium. Data are pooled from 4 independent experiments with n = 3–4 mice per experiment. Mean ± SEM, data were compared using mixed-effects analysis and Sidak's multiple comparisons test, ****p < 0.0001. Right panel: geometric mean fluorescence intensity of CD83 comparing the expression in LP versus epithelial CD103$^+$CD11b$^+$ DCs. Mean ± SEM, data are pooled from 3 independent experiments with n = 2–3 mice per experiment.

(E) Geometric mean fluorescence intensity of TNF$\alpha$ and IL-1$\beta$ measured by flow cytometry intracellular cytokine staining. Data are pooled from 3 independent experiments with n = 2–3 mice per experiment. For IL-1$\beta$, data were normalized by experiment. Data were compared using t test.

(F) Geometric mean fluorescence intensity of the costimulatory molecules CD86 and CD80 comparing the expression in CD103$^+$CD11b$^+$ DCs from epithelium and lamina propria. Data are pooled from 3 independent experiments, with n = 2–3 mice per experiment and were compared using Mann-Whitney test or t test.

(G) Left panel: geometric mean fluorescence intensity of CD209a comparing the expression in LP versus epithelial CD103$^+$CD11b$^+$ DCs. Data are pooled from 3 independent experiments normalized by experiment with n = 2–3 mice per experiment and compared using t test. Right panel: *Defa24* gene expression measured by quantitative real-time PCR. Fold change of epithelial over LP CD103$^+$CD11b$^+$ DCs expression normalized against the housekeeping *Hprt*. Data correspond to 3 independent experiments, with RNA obtained after pooling 4 mice per experiment. In (E)-(G), mean ± SEM, **p < 0.01, *** p < 0.001, **** p < 0.0001.

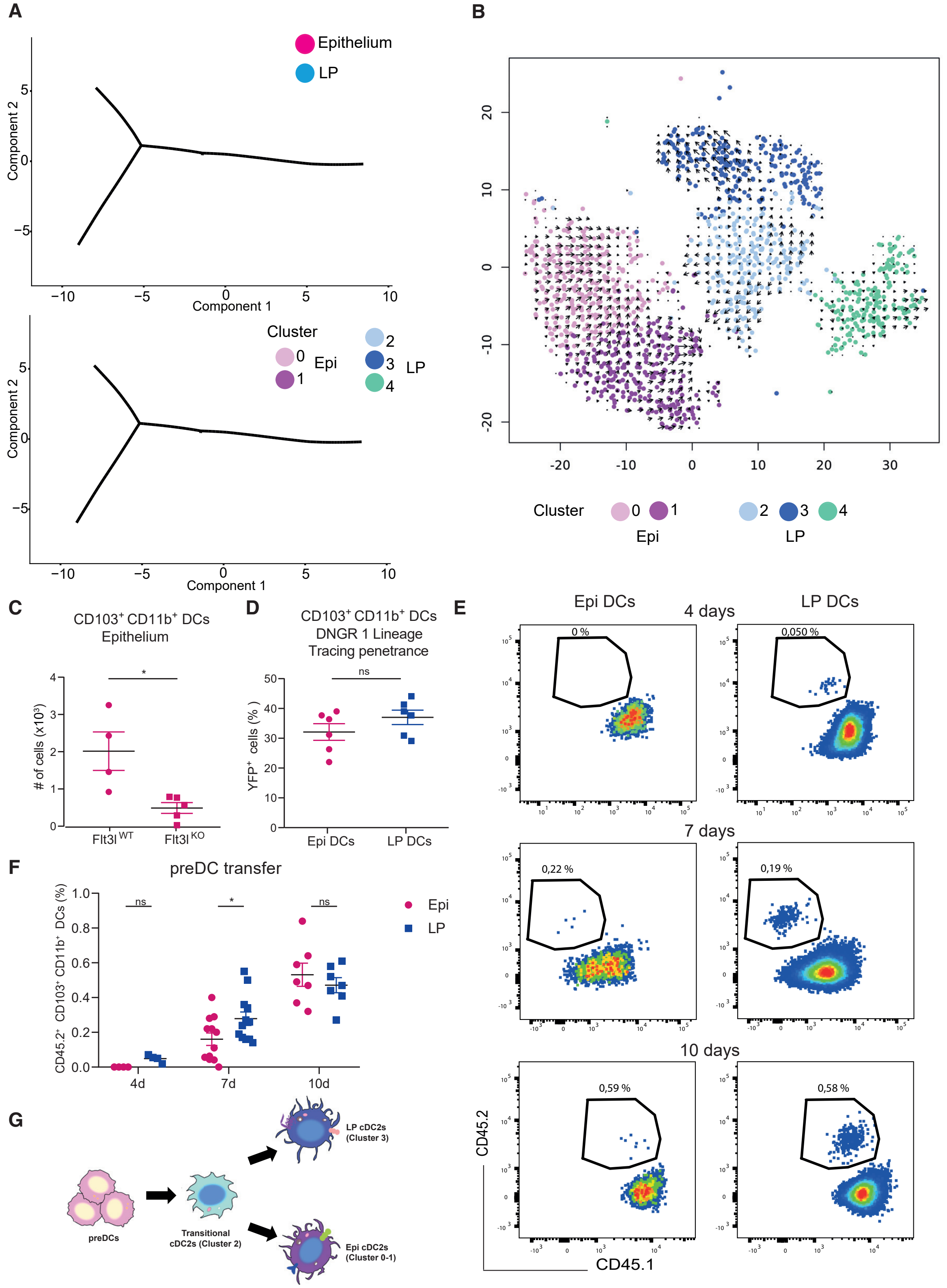


**Figure 3. LP and intraepithelial CD103$^+$CD11b$^+$ cDC2s originate from common pre-DC precursors**

(A) Pseudotime reconstruction of the trajectory analysis performed with monocle 2 based on all the differentially expressed genes of the unbiased clustering, by samples identification (top panel) and by cluster (bottom panel).

(legend continued on next page)

receptors *Pilra* and *Pilrb* genes and *Amica1* (Figures 2A and 4A) (Goswami et al., 2017; Zen et al., 2005), (2) actin nucleation and organization (GO Molecular Function 2017, p value 1.594e-3), and (3) cell response to mechanical stimuli (GO Biological Process 2017, p value 4,75e-4).

We thus tested the involvement of these three pathways. As to "transendothelial migration," we found fewer intraepithelial CD103$^+$CD11b$^+$ cDC2s in *Pilra*$^{-/-}$ mice (Figure 4B, left panel; Figure S2A), suggesting that this paired receptor might also be involved in transmigration through epithelia, at least in gut DCs. Concerning "actin nucleation," we assessed the involvement of ARPC4, an essential subunit of the Arp2/3 complex, as the expression of the *Abi3* gene (subunit of the WAVE complex), which activates branched actin nucleation by Arp2/3, was enriched in clusters 0-1 (Figure 4C, *Abi3*$^{-/-}$ being not available). As for *Pilra*, we found fewer intraepithelial DCs in the *Arpc4*$^{-/-}$ mice (*Arpc4*$^{flox/flox}$ *Itgax*$^{Cre}$, Figure 4D, left panel; Figure S2B). This result was not trivial, as it has been shown that bone-marrow-derived DCs do not require WAVE and Arp2/3 to migrate in microchannels and collagen gels (Vargas et al., 2016). It shows that the Arp2/3 complex was nonetheless required for epithelium colonization by LP CD103$^+$CD11b$^+$ cDC2s. Finally, regarding the "cell response to mechanical stimuli" pathway, we turned to myosin IIA, which is responsible for actomyosin contractility and the master regulator of DC migration in constrained environments (Chabaud et al., 2015; Lämmermann et al., 2008). Although *Myh9* (myosin IIA gene) was not as strongly upregulated as *Abi3* in intraepithelial cDC2s (Figures 4E and S2C), we observed a complete loss of intraepithelial cDC2s in myosin IIA-deficient mice (Figure 4F, left panel; Figure S2D).

Of note, while *Pilra*$^{-/-}$ and *Arpc4*$^{-/-}$ mice displayed normal numbers of LP CD103$^+$CD11b$^+$ cDC2s (Figure 4B, right panel, and Figure 4D, right panel), this was not the case in *Myh9*$^{-/-}$ animals, which displayed a ~40% decrease in this cell population (Figure 4F, right panel). No defect in the numbers of bone marrow DC precursors or LP cDC1s and CD103$^-$CD11b$^+$ cDC2s was observed in *Myh9*$^{-/-}$ mice (Figures S3A–S3D). These results suggest that, in addition to being needed for epithelial colonization by CD103$^+$CD11b$^+$ cDC2s, myosin IIA is also directly or indirectly involved in their development and/or survival. We conclude that epithelial colonization by CD103$^+$CD11b$^+$ LP cDC2s requires both branched actin and actomyosin contractility, in addition to transmigration-associated molecules such as the Pilrα receptor. They further highlight that transmigration into the epithelium is not required for survival of CD103$^+$CD11b$^+$ LP cDC2s as both *Pilra*$^{-/-}$ and *Arpc4*$^{-/-}$ cells were unable to colonize the epithelium but were present in normal numbers in the LP.

## Epithelial colonization by CD103$^+$CD11b$^+$ cDC2s requires ATRA

We next asked whether epithelial colonization by CD103$^+$CD11b$^+$ cDC2s was triggered by local environmental cues: the microbiota and/or the food-derived metabolites known to accumulate in the small intestine. Treatment of mice with antibiotics or antifungal agents did not alter the number of intraepithelial cDC2s (Figure 5A). As expected, we observed that the cecum of antibiotic-treated animals was considerably enlarged (Figure S4A), confirming the efficacy of these drugs. Hence, the microbiota does not influence epithelial colonization by cDC2s.

A good candidate metabolite to control epithelial colonization by CD103$^+$CD11b$^+$ cDC2s was vitamin A-derived all-*trans*-retinoic acid (ATRA), which preferentially accumulates in the upper region of the small intestine (Villablanca et al., 2011). Accordingly, we found that intraepithelial CD103$^+$CD11b$^+$ cDC2s were more abundant in the mouse duodenum than the jejunum, and almost totally absent in the ileum (Figure 5B, left panel). Although this difference was also observed for LP CD103$^+$CD11b$^+$ cDC2s, it did not reach statistical significance (Figure 5B, right panel). In addition, our single-cell RNA-seq results showed that expression of the *Spi1*(PU.1) transcription factor, which induces the expression of Aldh1a2, the enzyme responsible for ATRA production from retinal (Yashiro et al., 2018), was enriched in intraepithelial cDC2s compared with LP cDC2s (Figure 5C). Moreover, we found higher expression of the ATRA receptor alpha (RARα) in intraepithelial cDC2s than in their LP counterparts (Figure 5D).

To investigate the role of ATRA in the transmigration of cDC2s from the LP to the epithelium, we inhibited its production by treating mice with the retinaldehyde dehydrogenase 2 (RALDH2) inhibitor bisdiamine (Figure S4B). We found that this molecule reduced the number of intraepithelial cDC2s (Figure 5E, left panel). However, the number of LP cDC2s also diminished (Figure 5E, middle panel), which is consistent with previous results showing that ATRA is required to maintain CD103$^+$CD11b$^+$ cDC2s in the small intestine (Klebanoff et al., 2013). Nevertheless, we observed that the percentage of intraepithelial cDC2s was strongly decreased in bisdiamine-treated

(B) RNA velocity analysis of single-cell RNA-seq data showing that transitional cluster 2 might represent an intermediate cDC2 state between lamina propria cluster 3 and epithelial clusters 0 and 1.

(C) Plot of numbers of CD103$^+$CD11b$^+$ in live CD45$^+$ CD11c$^+$ MHCII$^+$ CD64$^-$ cells from the epithelium of the whole small intestine of *Flt3l*$^{-/-}$ and *Flt3l*$^{+/+}$ mice. Mean ± SEM, data are pooled from two independent experiments and compared using t test, *$p < 0.05$. Each symbol represents one mouse.

(D) Extent of DNGR 1 fate mapping measured as percentage of YFP$^+$ cells in CD103$^+$CD11b$^+$ cells (live CD45$^+$ CD11c$^+$ MHCII$^+$ CD64$^-$) from the lamina propria or epithelium of the whole small intestine of *Clec9a*$^{+/Cre}$Rosa$^{+/EYFP}$ mice. In total, n = 6 mice corresponding to one independent experiment. Mean ± SEM, data were compared using t test.

(E) Flow cytometry dot plots of CD45.2 pre-DC transfer experiments showing differentiation dynamics in the small intestine of syngeneic CD45.1 mice. CD45.2 mice were injected i.v. with pre-DCs sorted from BM of CD45.1 donor mice, and the differentiation into CD103$^+$CD11b$^+$ DCs was followed after 4, 7, and 10 days after transfer. Representative of 3 independent experiments.

(F) Quantification of pre-DC transfer experiments showing the percentage of donor-derived CD45.2$^+$ CD103$^+$CD11b$^+$ in live CD45$^+$ CD11c$^+$ MHCII$^+$ CD64$^-$ cells. Mean ± SEM, data are pooled from three independent experiments with n = 2–4 mice per time point per experiment. Data were compared using two-way ANOVA, *$p < 0.05$.

(G) Pre-DCs arriving from BM in the small intestinal lamina propria could differentiate first in transitional cluster 2 cDC2s, which constitute an intermediate state between lamina propria cDC2s (cluster 3) and intraepithelial cDC2s (clusters 0 and 1). Please also see Figure S1.

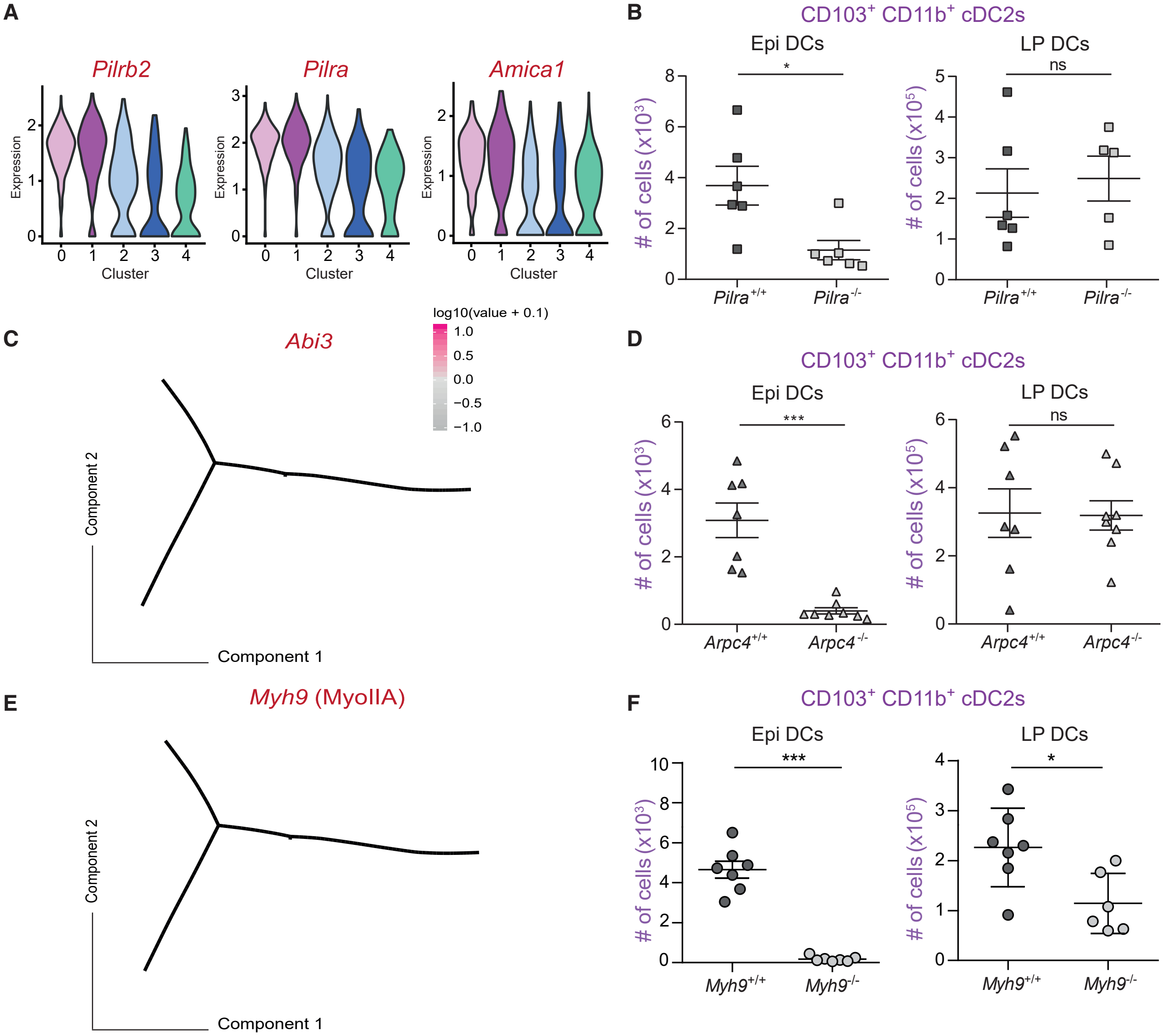


**Figure 4. The PILRα paired receptor and the actomyosin cytoskeleton are needed for epithelium colonization by CD103⁺CD11b⁺ cDC2s**

(A) Violin plot representing the expression of transendothelial migration-related genes among clusters of single-cell RNA-seq analysis: *Pilra*, *Pilrb*, and *Amica1*.

(B) Plots of number of $CD103^+CD11b^+$ in live $CD45^+$ $CD11c^+$ $MHCII^+$ $CD64^-$ cells from the epithelium and lamina propria of the whole small intestine in $Pilra^{-/-}$ and $Pilra^{+/+}$ female mice. Data are pooled from three independent experiments, each symbol represents one mouse.

(C) Pseudotime reconstruction showing the expression of the migratory related gene *Abi3*.

(D) Plots of number of $CD103^+CD11b^+$ in live $CD45^+$ $CD11c^+$ $MHCII^+$ $CD64^-$ cells from the epithelium and lamina propria of the whole small intestine in $Arpc4^{-/-}$ ($Arpc4^{flox/flox}$ × $Itgax^{Cre+}$) and $Arpc4^{+/+}$ ($Arpc4^{flox/flox}$ × $Itgax^{Cre-}$) mice. Data are pooled from three independent experiments, each symbol represents one mouse.

(E) Pseudotime reconstruction showing the expression of the migratory related gene *Myh9* (MyoIIA).

(F) Plots of number of $CD103^+CD11b^+$ in live $CD45^+$ $CD11c^+$ $MHCII^+$ $CD64^-$ cells from the epithelium and lamina propria of the whole small intestine in MyoIIA deficient ($Myh9^{flox/flox}$ × $Itgax^{Cre+}$) and WT ($Myh9^{flox/flox}$ × $Itgax^{Cre-}$) mice. Data are pooled from three independent experiments, each symbol represents one mouse. In (B), (D), and (F), mean ± SEM, data were compared using Mann-Whitney test or t test, *p < 0,05, ***p < 0,001. Please also see Figures S2 and S3.

mice (Figure 5E, right panel), suggesting that ATRA might also reduce epithelial colonization by cDC2s, in addition to their survival. A similar conclusion was reached when feeding mice with a diet free of vitamin A, from which ATRA is produced: while intraepithelial $CD103^+CD11b^+$ cDC2s were decreased in the duodenum of vitamin A-deprived mice (Figure 5F), their LP counterparts were not significantly affected (Figure S4C). Of note, lack of vitamin A did not abrogate the gradient formed by $CD103^+CD11b^+$ cDC2s along the intestine, suggesting the involvement of additional cues than ATRA in their compartmentalization. No significant difference was observed when treating mice with a vitamin A-supplemented diet. Altogether, these *in vivo* results suggest that ATRA might have an additional effect on intraepithelial cDC2s compared with the one it

CellPress
OPEN ACCESS

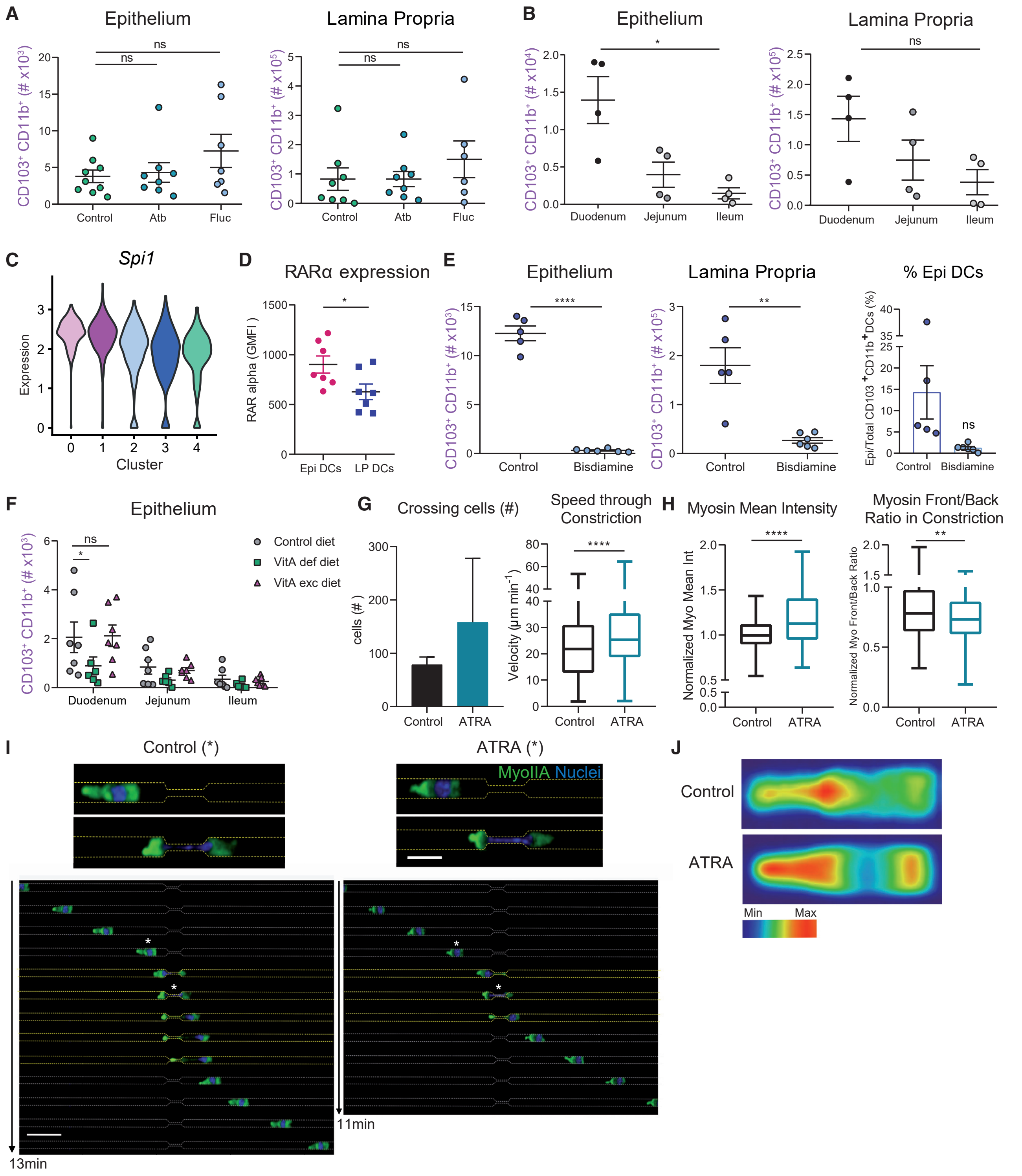


**Figure 5. CD103$^+$CD11b$^+$ cDC2s transmigration into the epithelium depends on ATRA**

(A) Adult SPF C57BL/6 mice were gavaged with PBS, antibiotic cocktail (Atb) or fluconazole (Fluc) for 10 days, and the numbers of CD103$^+$CD11b$^+$ cells in epithelium and lamina propria were determined by flow cytometry. Mean ± SEM, data are pooled from 3 independent experiments and compared using one-way ANOVA and Tukey's multiple comparisons test. Each symbol represents one mouse.

(B) Plots of number of CD103$^+$CD11b$^+$ in live CD45$^+$ CD11c$^+$ MHCII$^+$ CD64$^-$ cells from the epithelium (left panel) or lamina propria (right panel) of small intestinal duodenum, jejunum, and ileum. Data are pooled from two independent experiments. Mean ± SEM, data were compared using Kruskal-Wallis test or paired one-way ANOVA and Tukey's multiple comparisons test, *$p < 0.05$. Each symbol represents one mouse.

(legend continued on next page)

has on global cDC2 survival. However, they prevent us from reaching a formal conclusion on the involvement of this food-derived metabolite in transmigration and epithelial colonization by cDC2s.

To obtain direct evidence of this, we thus turned to an *in vitro* strategy. We sorted LP cDC2s, treated them or not with ATRA, and let them migrate into microfabricated channels containing small constrictions (1.5–2 μm). For these experiments, we used LP cDC2s from green fluorescent protein (GFP)-tagged *Myh9* mice to assess the effect of ATRA on the actin motor. We found that the percentage of cells able to migrate through constrictions was considerably increased by the treatment, suggesting that ATRA promoted their passage through small holes (Figure 5G, left panel). Furthermore, the speed of cDC2s passage through constrictions was enhanced by ATRA (Figure 5G, right panel), indicating that this metabolite does indeed increase the capacity of cDC2s to migrate in confined environments. Additionally, we observed that ATRA increased GFP-*Myh9* expression in migrating cDC2s as well as the accumulation of this motor protein at the cell rear (Figures 5H–5J) (Bretou et al., 2017), indicating that it enhanced actomyosin contractility. Altogether these results strongly suggest that, besides its *in vivo* role in maintaining $CD103^+CD11b^+$ cDC2 numbers in the small intestine, ATRA further stimulates myosin IIA-dependent contractility, transmigration, and epithelium colonization by these cells.

## The immature phenotype of intraepithelial cDC2s results from epithelium imprinting

Our results so far suggest that transmigration of cDC2s into the small intestine epithelium endows them with an immature-like DC phenotype. We next investigated the mechanisms involved in the acquisition of this phenotype by intraepithelial DCs. Two nonexclusive mechanisms could account for it: (1) transmigration through the basement membrane that separates the LP from the epithelium could per se shape the phenotype of cDC2s, analogously to what was proposed for DC differentiation from monocytes (Randolph et al., 1998); (2) this phenotype could result from exposure to local epithelial cues, consistent with a previous report showing that incubation of human monocyte-derived DCs with the Caco2 epithelial cell line *in vitro* can enhance their tolerogenic potential (Iliev et al., 2009a). To test these hypotheses, we used a transwell assay where sorted LP $CD103^+CD11b^+$ cDC2s were allowed to migrate through 3 μm pores from the upper to the lower compartment. We found that migration through 3 μm pores had no impact on the phenotype of these cells, as shown by their CCR7 and CD86 surface expression (Figure 6A). In contrast, surface expression of these molecules was significantly downregulated when coculturing LP $CD103^+CD11b^+$ cDC2s with epithelial cells (Figures 6B, 6C, and S5A). These data, therefore, suggest that the immature phenotype of intraepithelial cDC2s does not result from their transmigration per se but is rather imprinted by epithelial cues.

## The immature phenotype of intraepithelial cDC2s relies on both ATRA and Muc2

To gain an insight into the epithelial cues involved, we prepared gut organoids from the duodenum (Figure 6D). Incubation of sorted LP cDC2s with these organoids strongly downregulated their CCR7 surface expression (Figures 6E and S5B). In contrast, the expression of CD86 did not decrease. Of note, LP cDC2s were recruited and allowed to physically interact with these duodenal 3D structures embedded into Matrigel. However, they did not penetrate their lumen (Figure 6D), suggesting that transmigration was not very effective in this experimental system. These data suggest that while CCR7 might be downregulated by soluble cues that diffuse out from organoids, CD86 might rather respond to apical cues present in their lumen and to which sorted cDC2s cannot access in the context of organoids. These results indicate that diverse epithelial cues might be acting together to imprint the immature phenotype of intraepithelial cDC2s.

We turned to the literature to identify potential cues involved. Among the soluble cues described was transforming growth

(C) Violin plot showing the expression of *Spi1* gene among clusters of single-cell RNA-seq analysis of $CD103^+CD11b^+$ DCs. Cluster 0 versus 3: p value 3,11E-11; cluster 1 versus 3: p value 2,84E-18.

(D) Retinoic acid receptor alpha expression measured by intracellular staining of $CD103^+CD11b^+$ DCs comparing epithelium and lamina propria. Mean ± SEM, data are pooled from 3 independent experiments with n = 2–3 per experiment and compared using t test, $^*p < 0.05$.

(E) Adult SPF C57BL/6J mice were gavaged with olive oil or bisdiamine for 2 days. Plots represent the number of $CD103^+CD11b^+$ cells in the epithelium (left) and lamina propria (middle) from the entire small intestine determined by flow cytometry. Mean ± SEM, data are pooled from 2 independent experiments and representative of 3 independent experiments. Each symbol represents one mouse. Data were compared using t test, $^{**}p < 0.01$, $^{****}p < 0.0001$. Right panel: percentage of intraepithelial DCs among total $CD103^+CD11b^+$ dendritic cells in control or bisdiamine-treated mice.

(F) Flow cytometry analysis of $CD103^+CD11b^+$ DC numbers from the small intestinal epithelium analyzed in duodenum, jejunum, and ileum of SPF C57BL/6J mice fed with vitamin A-deficient, excess vitamin A, or control diet for 3 months. Mean ± SEM, data are pooled from 2 independent experiments and compared using two-way ANOVA, $^*p < 0.05$. Each symbol represents one mouse.

(G) Quantification of cells inside microchannels of 4 × 4 μm crossing constrictions of 1.5–2 μm. Lamina propria $CD103^+CD11b^+$ DCs were sorted from GFP-tagged *Myh9* mice and let them migrate inside microchannels with constrictions overnight. Control $CD103^+CD11b^+$ DCs or $CD103^+CD11b^+$ DCs treated with 1 nM ATRA were included in the experiment (left panel). Speed of lamina propria $CD103^+CD11b^+$ DCs while migrating into the constrictions measured as μm/min (right panel).

(H) Quantification of myosine IIA mean intensity of lamina propria $CD103^+CD11b^+$ DCs while migrating through microchannels. Values were normalized against control average (left panel). Front/back ratio of myosin IIA distribution inside $CD103^+CD11b^+$ LP DCs while migrating through microchannels (right panel). In (G) and (H), mean ± SEM, data are pooled from 2 independent experiments. Total number of cells analyzed: 157 cells (control), 316 (ATRA). Data were compared using Mann-Whitney test, $^{**}p < 0.01$, $^{****}p < 0.0001$.

(I) Upper panels: zoom-in of $CD103^+CD11b^+$ LP DCs migrating intro microchannels and passing through constrictions of between 1.5 and 2μm for control and ATRA conditions. Scale bar, 40 μm.

Lower panels: montage of $CD103^+CD11b^+$ LP DCs migrating into microchannels. White stars show $CD103^+CD11b^+$ DCs zoomed-in on the upper panels. Scale bar, 100 μm.

(J) Heatmap of myosin IIA distribution of LP $CD103^+CD11b^+$ DCs passing through the constrictions. Please also see Figure S4.

CellPress
OPEN ACCESS

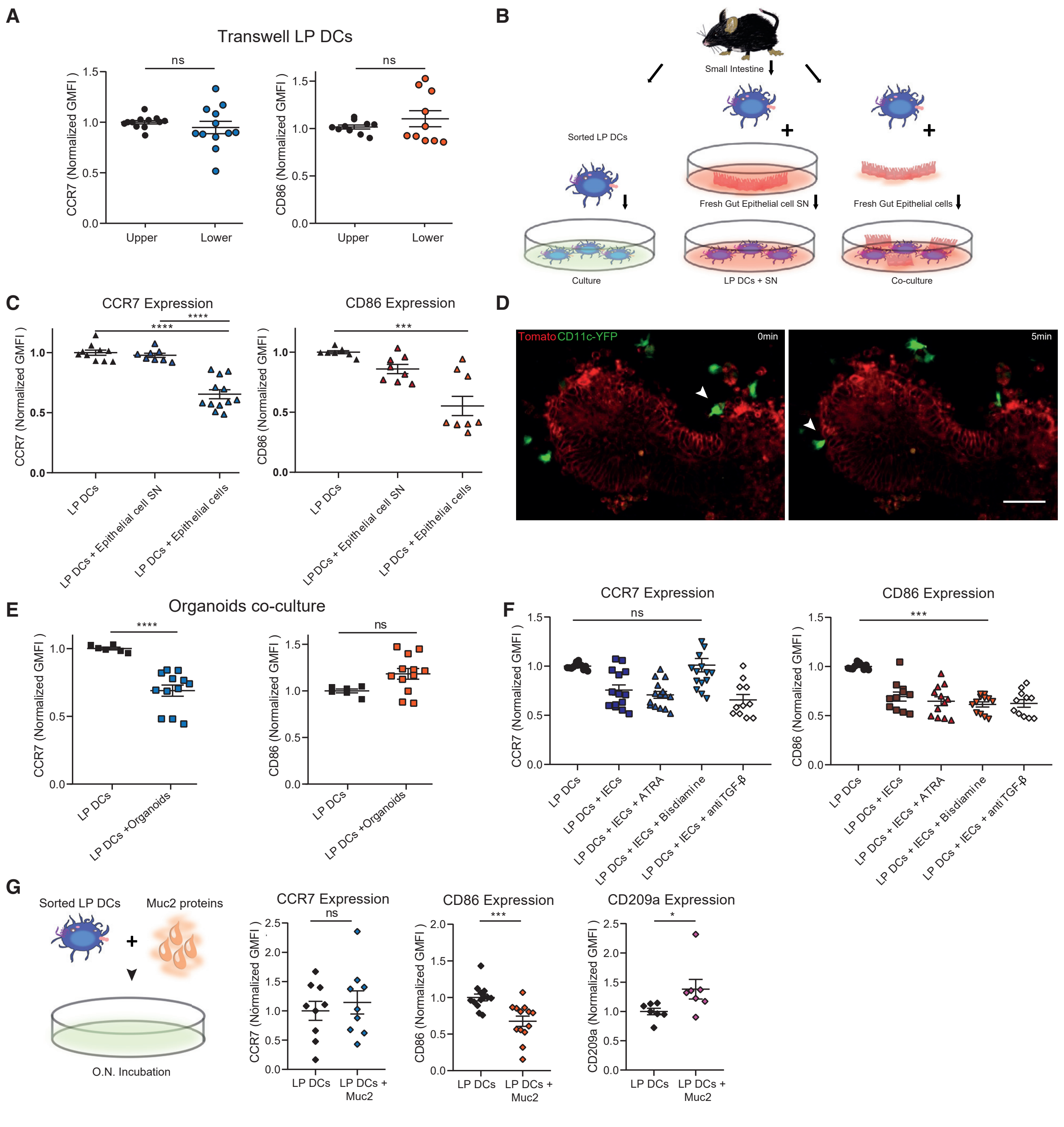


**Figure 6. Epithelial colonization imprints CD103$^+$CD11b$^+$ cDC2s with an immature-like phenotype**

(A) Transwell experiments were performed with purified lamina propria CD103$^+$CD11b$^+$ DCs, and geometric mean fluorescence intensity of CCR7 and CD86 was analyzed by flow cytometry after overnight transmigration. DCs transmigrated from the upper to the lower compartment passing through pores of 3 μm. Mean ± SEM, data are pooled from 3 independent experiments and compared using t test.

(B) Freshly obtained SI epithelial cells were obtained from the small intestine of C57BL/6J mice, as well as sorted lamina propria CD103$^+$CD11b$^+$ DCs. Gut epithelial cell supernatant (SN) was obtained after 6 h of incubation at 37°C with 5% $CO_2$. Sorted LP CD103$^+$CD11b$^+$ DCs were either incubated alone, with gut epithelial cells or their supernatant overnight. Differentially expressed markers were analyzed by flow cytometry.

(C) Geometric mean fluorescence intensity of CCR7 and CD86 comparing LP CD103$^+$CD11b$^+$ DCs incubated with small intestine epithelial cells, supernatant of small intestine epithelial cells, or control LP CD103$^+$CD11b$^+$ DCs alone. Mean ± SEM, data are pooled from 3 independent experiments and compared using one-way ANOVA or Kruskal-Wallis test, ***$p < 0.001$, ****$p < 0.0001$.

(D) Small intestinal duodenal organoids were derived from a membrane-fluorescent mice reporter, and after 4 days of culture, LP CD103$^+$CD11b$^+$ DCs purified from *Itgax*-EYFP reporter mice were added to the culture. Images of the interaction between DCs and organoids. Scale bar, 80 μm. Representative of two independent experiments.

(legend continued on next page)

factor β (TGF-β), which can be secreted by epithelial cells and endow mouse bone marrow DCs with anti-inflammatory properties *ex vivo* (Iliev et al., 2009b). Addition of blocking anti-TGF-β antibodies to cocultures including epithelial cells and sorted LP CD103$^+$CD11b$^+$ cDC2s did not affect their phenotype, suggesting that this cytokine might not play an essential role in this process *in vivo*. ATRA is also known for its tolerogenic action, in particular through the induction of Treg cells. We thus tested its effect on the cDC2 phenotype and found that inhibition of ATRA by bisdiamine restored CCR7 expression without affecting the expression of CD86 (Figures 6F and S5C). Supplementing cocultures with ATRA had no significant effect, suggesting that the endogenous amounts of this metabolite are sufficient for it to reach its maximal effect on CCR7 expression. These results indicate that ATRA, which can be produced by both immune and epithelial cells, not only facilitates epithelial colonization by cDC2s but might also be part of the environmental cues that imprint these cells with an immature phenotype.

Concerning the insoluble epithelial cues that may contribute to the immature phenotype of intraepithelial cDC2s, we tested the potential involvement of the mucus produced by small intestine goblet cells. Indeed, it was reported that myeloid cells, including DCs, can internalize the mucus, which leads to downregulation of their proinflammatory properties (Shan et al., 2013). Remarkably, incubation of LP CD103$^+$CD11b$^+$ cDC2s with mucus protein 2 (Muc2) decreased their CD86 surface expression but did not affect CCR7 (Figure 6G). We further found that Muc2 enhanced the expression of the c-type lectin CD209a, which is indeed higher in intraepithelial than in LP cDC2s. Altogether these results show that the immature phenotype of intraepithelial cDC2s results from the concerted action of environmental cues: ATRA that downregulates CCR7, and Muc2, which diminishes the expression of CD86 while upregulating the one of CD209a.

### Intraepithelial cDC2s, but not LP cDC2s, exhibit tolerogenic properties

Finally, we investigated whether the acquisition of an immature phenotype by intraepithelial cDC2s translates into functional differences, compared with their LP counterparts. For this, we analyzed their antigen presentation capacities. CD103$^+$CD11b$^+$ cDC2s from the epithelium or the LP were sorted, preincubated with increasing concentrations of full-length ovalbumin (OVA) or OVA peptide (OVAp), and co-cultured with OT-II T cells. We found that LP cDC2s activated T cells efficiently, as shown by the upregulation of CD69, T cell proliferation, and interleukin-2 (IL-2) production (Figures 7A and 7B), which is consistent with their mature phenotype. In contrast, intraepithelial cDC2s were considerably less efficient at promoting T cell activation, even though they exhibited similar survival rates compared with LP cDC2s in cocultures (Figure S6A). Moreover, incubation of OT-II T cells that had previously interacted with intraepithelial cDC2s with anti-CD3 and CD28 antibodies led to upregulation of CD69 but failed to induce T cell proliferation (Figure 7C), indicative of a T cell hyporesponsive state. These results suggest that intraepithelial cDC2s induce T cell anergy rather than activation, highlighting their tolerogenic potential. These differences did not result from impaired antigen capture, as intraepithelial cDC2s showed higher OVA internalization than their LP counterparts (Figure 7D). Accordingly, we observed the presence of vesicles resembling macropinosomes in intraepithelial cDC2s (Figure 7E), supporting the idea that they display an immature-like phenotype.

Consistent with these findings, we found that intraepithelial cDC2s not only displayed lower expression of costimulatory molecules CD80 and CD86 than LP cDC2s but also expressed increased amounts of the inducible co-stimulatory molecule ligand (ICOS-L), which has been associated with the induction of tolerance (Figure 7F; Hubo et al. 2013). Of note, antibodies blocking ICOS-L were not sufficient to increase T cell activation or proliferation by intraepithelial cDC2s (Figure S6B), suggesting the involvement of additional costimulatory and/or cytokines in this process. Anyhow, these results provide direct experimental evidence for intraepithelial cDC2s having immature features, with low surface expression of CCR7 and costimulatory molecules, elevated antigen capture but decreased antigen presentation capacities, and the ability to promote T cell hyporesponsiveness. Together, our results highlight the existence of gut subtissular niches that can shape the identity of cDCs and profoundly modify their antigen presentation function.

## DISCUSSION

We here show that epithelial colonization of cDC2s in the small intestine under homeostatic conditions contributes to the functional diversification of this cDC subset by promoting the formation of two functionally distinct pools of cells: (1) a CD103$^+$CD11b$^+$ cDC2 pool that resides in the LP, which exhibits a "mature-like" proinflammatory phenotype and is able to migrate to lymph nodes (CCR7$^+$), and (2) a CD103$^+$CD11b$^+$ cDC2 pool located within the epithelium, which displays an "immature-like" phenotype and tolerogenic properties.

How do LP and intraepithelial cDC2s relate to each other? We found that intraepithelial CD103$^+$CD11b$^+$ cDC2s, as LP cDC2s, relied on the presence of Flt-3 ligand and expressed the same amounts of DNGR1, suggesting that they might differentiate from common pre-cDC precursors. These precursor cells move into the small intestine from blood vessels that are located within the LP. This implies that the precursor of intraepithelial cDC2s must maneuver through the LP before reaching their niche inside the epithelium (Farache et al., 2013), in agreement with our pre-DC transfer experiments and cell trajectory

(E) Geometric mean fluorescence intensity of CCR7 and CD86 comparing LP CD103$^+$CD11b$^+$ DCs incubated with organoids derived from duodenum or control LP cDC2s. Mean ± SEM, data are pooled from 3 independent experiments and compared using t test, ****$p < 0.0001$.
(F) Sorted LP CD103$^+$CD11b$^+$ DCs were incubated alone or with freshly obtained gut epithelial cells overnight, with either ATRA 1 nM, bisdiamine 45 μM, or anti-TGF-β 10 μg/ml. Differentially expressed markers were analyzed by flow cytometry, and geometric mean fluorescence intensity of CCR7 and CD86 is represented. Mean ± SEM, data are pooled from 3 independent experiments and compared using one-way ANOVA and Tukey multiple comparisons test, ***$p < 0.001$.
(G) Sorted LP CD103$^+$CD11b$^+$ DCs were incubated overnight with the mucus protein Muc2 (50 μg/ml), and CCR7, CD86, and CD209a expression was analyzed by flow cytometry. Mean ± SEM, data are pooled from 3 independent experiments (CCR7 and CD86) or two independent experiments (CD209a). Data were compared using t test (***$p < 0.001$) or Mann-Whitney test (*$p < 0.05$). Please also see Figure S5.

CellPress
OPEN ACCESS

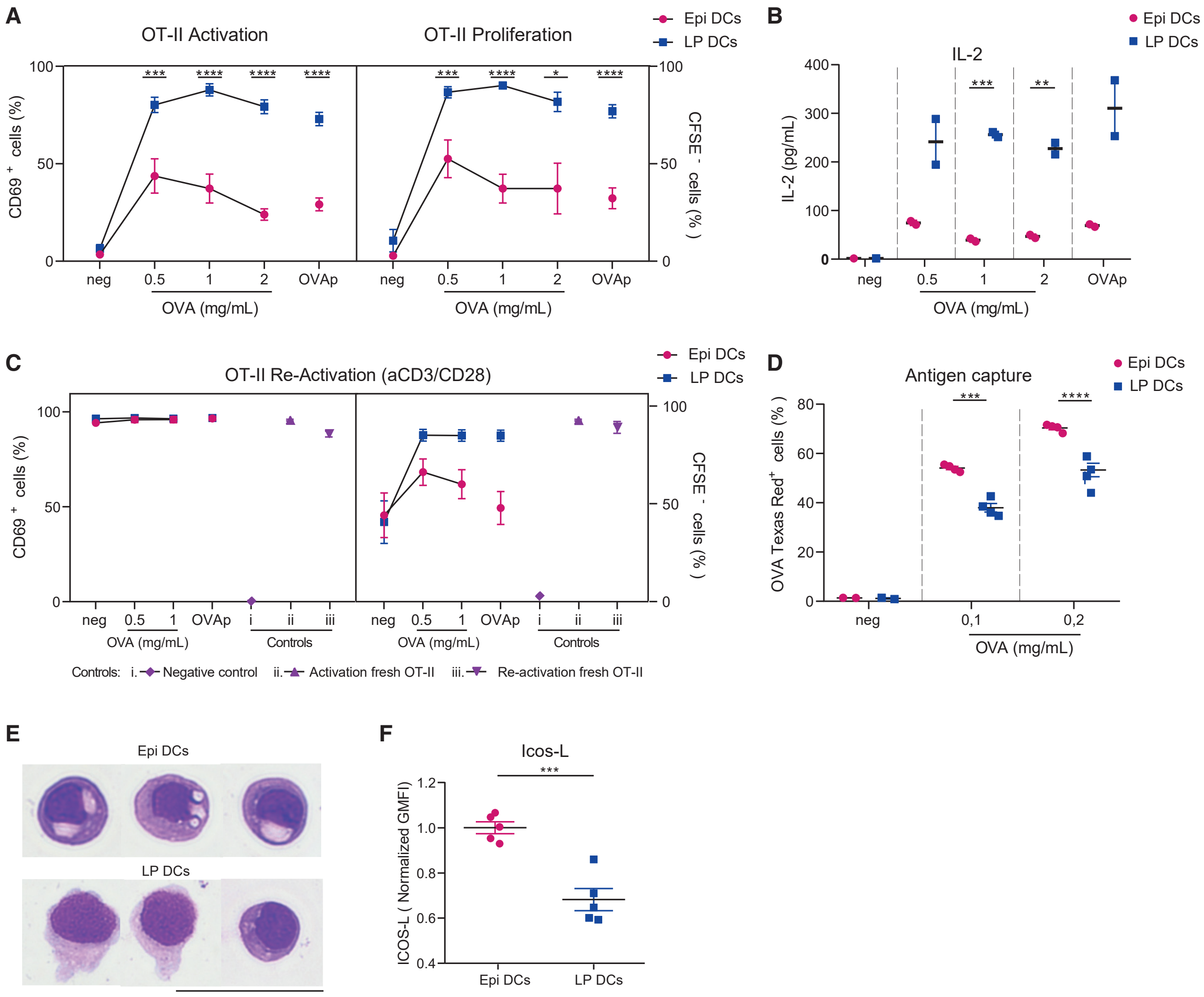


**Figure 7. Intraepithelial CD103$^+$CD11b$^+$ cDC2s are endowed with tolerogenic properties**

(A) Left panel: percentage of CD69 positive CD4$^+$ T cells in live cells after 18 h of incubation with OVA preincubated CD103$^+$CD11b$^+$ dendritic cells. Right panel: percentage of CFSE$^-$ CD4$^+$ T cells in live cells after 3 days of incubation with OVA or OVA peptide II preincubated CD103$^+$CD11b$^+$ dendritic cells, analyzed by flow cytometry. OT-II antigen presentation assay was performed with sorted CD103$^+$CD11b$^+$ DCs from both small intestine lamina propria and epithelium from C57BL/6J mice. Mean ± SEM, data are pooled from five independent experiments and compared using multiple t test or Mann-Whitney test, $^*p < 0.05$, $^{***}p < 0.001$, $^{****}p < 0.0001$.

(B) IL-2 secretion by OT-II T cells measured by Luminex. Representative of 2 independent experiments. Mean ± SEM, data were compared using Sidak's multiple comparisons test, $^{**}p < 0.01$, $^{***}p < 0.001$.

(C) CD4$^+$ OT-II T cells were incubated overnight with CD103$^+$CD11b$^+$ DCs (previously incubated with OVA or OVAp). T cells were incubated with anti-CD3 and anti-CD28 antibodies, and the percentage of CD69 positive cells (left panel) or CFSE negative cells (right panel) was measured after overnight or 2 days, respectively. Data are pooled from four independent experiments. Fresh OT-II T cells preactivated or not with anti-CD3 and anti-CD28 antibodies were used as controls (two independent experiments).

(D) Plot of percentage of OVA-Texas Red positive cells after incubation of sorted CD103$^+$CD11b$^+$ DCs from lamina propria and epithelium with fluorescent OVA, measured by flow cytometry. Mean ± SEM, data are pooled from three independent experiments and compared using two-way ANOVA and Sidak's multiple comparisons test, $^{***}p < 0.001$, $^{****}p < 0.0001$.

(E) Images of cytospin and MGG staining of CD103$^+$CD11b$^+$ DCs from lamina propria and epithelium after sorting. Representative of 2 independent experiments.

(F) Geometric mean fluorescence intensity of the costimulatory molecule ICOS-L comparing the expression in CD103$^+$CD11b$^+$ DCs from epithelium and lamina propria. Mean ± SEM, data are pooled from two independent experiments, normalized by experiment, and compared using t test, $^{***}p < 0.001$. Please also see Figure S6.

analyses. Notably, a recent report shows the presence of proliferative cells inside the human LP, with pre-DC features that correlate with the gene expression profile of our cycling cluster 4 (Fenton et al., 2021). Although RNA velocity analysis did not reveal a direct link between cluster 4 and the other cDC2 clusters, we cannot exclude that at least some of the cycling cells

contained in cluster 4 might contribute to the generation of gut CD103$^+$CD11b$^+$ cDC2s.

Could other subsets than cDC2s contribute to the formation of the intraepithelial DC pool through cell plasticity? CLEC9A fate mapping experiments strongly suggest that intraepithelial cDC2s are unrelated to LP cDC1s, which is consistent with no intraepithelial cDC1s being found at any time point in pre-DC transfer experiments. Regarding CD103$^-$CD11b$^+$ cDC2s, they were also absent from the pool of intraepithelial DCs, even at early time points upon pre-DC transfer, arguing against their contribution to the intraepithelial DC population. In agreement with this result, we found that intraepithelial cDC2s did not rely on the presence of CCR2. Indeed, this chemokine receptor was previously shown to participate in the formation of the gut CD103$^-$CD11b$^+$ cDC2 pool (Scott et al., 2015). Altogether, these results strongly suggest that intraepithelial cDCs are mainly related to LP CD103$^+$ CD11b$^+$ cDC2s rather than to other LP DC subsets.

Which specific needs could immature intraepithelial cDC2s fill in the upper region of the small intestine? First, they might allow the expansion of Foxp3$^+$ Treg cells, which, after acquiring gut homing markers in the mesenteric lymph nodes, migrate to the small intestine and undergo a secondary expansion that enables them to produce interleukin-10 (IL-10) (Cassani et al., 2011; Hadis et al., 2011). Second, intraepithelial cDC2s might be needed to convert Foxp3$^+$ CD4$^+$ cells into intraepithelial lymphocytes (IELs), which occurs upon Foxp3$^+$ Tregs transmigration into the epithelial cell layer (Sujino et al., 2016). In line with this hypothesis, it has been shown that DCs can influence IEL homeostasis (Luda et al., 2016). Third, intraepithelial cDC2s might simply limit the activity of IELs or act as a sink for food antigens or microbiome species that are abundant in the upper region of the intestine where intraepithelial cDC2s reside. They might uptake and locally process these microbes, limiting their spread to other organs as they lack CCR7 expression at steady state.

How is the phenotype and function of CD103$^+$CD11b$^+$ cDC2s modified upon epithelial colonization? Our results identified ATRA as an important regulator of this process: ATRA stimulated actomyosin contractility, then promoted cDC2 transmigration and epithelial colonization, and at the same time, it acted as an environmental cue, shaping the phenotype of these cells. Once cDC2s have reached the epithelium, the mucus protein Muc2 can decrease the surface expression of CD86 while increasing the expression of CD209a, which might help their antigen capture function. These data suggest the involvement of distinct imprinting cues for gut intraepithelial cDC2s to acquire their immature phenotype, with some of them having additive effects. They strongly suggest that the complexity of tissue-resident cDC populations most likely results from the concerted action of multiple cues to which they have access by physically reaching specific subtissular niches. Identifying these niches and cues within different tissues will thus be essential to understand cDC functional diversification and develop effective strategies to manipulate these cells in a pathological context.

## Limitations of the study

Due to technical limitations, we were unable to determine whether intraepithelial cDC2s constitute a pure gut-resident population or whether they can migrate to lymph nodes upon stimulation. It has been reported that *Salmonella* can trigger CCR7 expression at the surface of intraepithelial cDC2s *ex vivo* (Farache et al., 2013), but whether this occurs *in vivo* or upon contact with nonpathogenic bacteria is unknown. Defining whether intraepithelial cDC2s are migratory or not would help in understanding their physiological function both at steady state and upon infection. Generation of mouse models that allow specific inhibition of epithelial colonization by cDC2s without affecting their migration to lymph nodes should help addressing these questions.

## STAR★METHODS

Detailed methods are provided in the online version of this paper and include the following:

- KEY RESOURCES TABLE
- RESOURCE AVAILABILITY
  - Lead contact
  - Materials availability
  - Data and code availability
- EXPERIMENTAL MODEL AND SUBJETC DETAILS
  - Mice
- METHODS DETAILS
  - Isolation of Intestinal Cell Suspensions
  - Flow cytometry analysis
  - Single-cell RNA sequencing
  - Tissue immunofluorescence
  - Quantitative real-time RT-PCR
  - Adoptive transfer of preDCs
  - EdU turnover rate analysis
  - Immunoblotting
  - Antibiotic and anti-fungal treatment
  - Inhibition of RALDH activity
  - Micro-channels preparation and analysis
  - Transwell experiments
  - DC co-culture with epithelial cells and mucus
  - Organoid-DC co-culture
  - Antigen presentation and capturing assay
  - Cytospin and MGG staining
- QUANTIFICATION AND STATISTICAL ANALYSIS

## SUPPLEMENTAL INFORMATION

Supplemental information can be found online at https://doi.org/10.1016/j.immuni.2021.11.008.

## ACKNOWLEDGMENTS

We thank Institut Curie for access to the flow cytometry, animal, and the cell and tissue imaging facilities (PICT-IBiSA). We thank Emmanuel Gautier for kindly providing Ccr2$^{-/-}$ mice. C.A.R. thanks Gehenna Guerrero-Serrano for teaching her how to prepare gut organoids and the ARC foundation for financial support. This project has received funding from INSERM, Institut Curie, and the DCBIOL Labex (ANR-10-IDEX-0001-02-PSL and ANR-11-LABX-0043). High-throughput sequencing was performed by the ICGex NGS platform of the Institut Curie supported by the grants ANR-10-EQPX-03 (Equipex) and ANR-10-INBS-09-08 (France Génomique Consortium) from the Agence Nationale de la Recherche ("Investissements d'Avenir" program), by the ITMO-Cancer Aviesan (Plan Cancer III), and by the SiRIC-Curie program (SiRIC grant INCa-DGOS-4654). Research in the C.R.S. laboratory is supported by The Francis Crick Institute, which receives core funding from Cancer Research UK (FC001136), the UK Medical Research Council (FC001136), and the

CellPress
OPEN ACCESS

Wellcome Trust (FC001136), by an ERC Advanced Investigator grant (AdG 268670), by a Wellcome Investigator Award (WT106973/Z/15/Z), and by a prize from the Louis-Jeantet Foundation. Our research is funded in whole, or in part, by the Wellcome Trust (grants FC001136 and 106973/Z/15/Z). For the purpose of Open Access, the author has applied a CC BY public copyright licence to any Author Accepted Manuscript version arising from this submission.

## AUTHOR CONTRIBUTION

C.A.R. conceived and performed most of the experimental work and actively contributed to manuscript preparation by building figures and writing part of the manuscript. V.R. initiated the project when observing the impairment of Myh9$^{-/-}$ DCs to migrate into the epithelium and performed antibiotic and bisdiamine treatment experiments. W.R. and C.G. performed bioinformatics analyses. Y.G.-F. helped C.A.R. with pre-DC transfer experiments. M.-G.D. helped C.A.R. performing microchannel experiments and analyzed the data obtained together with M.M. A.S.C. helped setting up tissue imaging experiments. A.F., C.S., and S.M.P. performed experiments with vitamin A-deficient food and provided their expertise in gut flow cytometry analysis. H.K.-C. and M.L. helped in designing and performing mucus-related experiments. S.L. and S.B. prepared RNA-seq libraries. P.G. provided key mouse tools and conceptual advice. M.C.-C. and C.R.S. helped C.A.R. in designing and performing Clec9A experiments. J.H. helped in designing pre-DC transfer experiments and provided conceptual advice. H.D.M., D.M.V., and E.J.V. provided essential intellectual input throughout the project. A.-M.L.-D. supervised this study and wrote the manuscript. All authors contributed to manuscript preparation.

## DECLARATION OF INTERESTS

C.R.S. has an additional appointment as professor in the Faculty of Medicine at Imperial College London. C.R.S. is a founder of Adendra Therapeutics and owns stock options and/or is a paid consultant for Adendra Therapeutics, Bicara Therapeutics, Montis Biosciences, Oncurious NV, Bicycle Therapeutics, and Sosei Heptares, all unrelated to this work.

Received: June 15, 2021
Revised: August 19, 2021
Accepted: November 15, 2021
Published: December 14, 2021

## REFERENCES

Bogunovic, M., Ginhoux, F., Helft, J., Shang, L., Hashimoto, D., Greter, M., Liu, K., Jakubzick, C., Ingersoll, M.A., Leboeuf, M., et al. (2009). Origin of the lamina propria dendritic cell network. Immunity *31*, 513–525.

Bretou, M., Sáez, P.J., Sanséau, D., Maurin, M., Lankar, D., Chabaud, M., Spampanato, C., Malbec, O., Barbier, L., Muallem, S., et al. (2017). Lysosome signaling controls the migration of dendritic cells. Sci. Immunol. *2*, eaak9573.

Cabeza-Cabrerizo, M., Cardoso, A., Minutti, C.M., Pereira da Costa, M., and Reis e Sousa, C. (2021). Dendritic cells revisited. Annu. Rev. Immunol. *39*, 131–166.

Cabeza-Cabrerizo, M., van Blijswijk, J., Wienert, S., Heim, D., Jenkins, R.P., Chakravarty, P., Rogers, N., Frederico, B., Acton, S., Beerling, E., et al. (2019). Tissue clonality of dendritic cell subsets and emergency DCpoiesis revealed by multicolor fate mapping of DC progenitors. Sci. Immunol. *4*, eaaw1941.

Cassani, B., Villablanca, E.J., Quintana, F.J., Love, P.E., Lacy-Hulbert, A., Blaner, W.S., Sparwasser, T., Snapper, S.B., Weiner, H.L., and Mora, J.R. (2011). Gut-tropic T cells that express integrin α4β7 and CCR9 are required for induction of oral immune tolerance in mice. Gastroenterology *141*, 2109–2118.

Castillo, P.A., Nonnecke, E.B., Ossorio, D.T., Tran, M.T.N., Goley, S.M., Lönnerdal, B., Underwood, M.A., and Bevins, C.L. (2019). An experimental approach to rigorously assess Paneth cell α-defensin (Defa) mRNA expression in C57BL/6 mice. Sci. Rep. *9*, 13115.

Caton, M.L., Smith-Raska, M.R., and Reizis, B. (2007). Notch-RBP-J signaling controls the homeostasis of CD8-dendritic cells in the spleen. J. Exp. Med. *204*, 1653–1664.

Chabaud, M., Heuzé, M.L., Bretou, M., Vargas, P., Maiuri, P., Solanes, P., Maurin, M., Terriac, E., Le Berre, M., Lankar, D., et al. (2015). Cell migration and antigen capture are antagonistic processes coupled by myosin II in dendritic cells. Nat. Commun. *6*, 7526.

Chen, E.Y., Tan, C.M., Kou, Y., Duan, Q., Wang, Z., Meirelles, G.V., Clark, N.R., and Ma'ayan, A. (2013). Enrichr: interactive and collaborative HTML5 gene list enrichment analysis tool. BMC Bioinformatics *14*, 128.

Chikina, A.S., Nadalin, F., Maurin, M., San-Roman, M., Thomas-Bonafos, T., Li, X.V., Lameiras, S., Baulande, S., Henri, S., Malissen, B., et al. (2020). Macrophages maintain epithelium integrity by limiting fungal product absorption. Cell *183*, 411–428, e16.

Dobin, A., Davis, C.A., Schlesinger, F., Drenkow, J., Zaleski, C., Jha, S., Batut, P., Chaisson, M., and Gingeras, T.R. (2013). STAR: ultrafast universal RNA-seq aligner. Bioinformatics *29*, 15–21.

Esterházy, D., Loschko, J., London, M., Jove, V., Oliveira, T.Y., and Mucida, D. (2016). Classical dendritic cells are required for dietary antigen-mediated induction of peripheral T(reg) cells and tolerance. Nat. Immunol. *17*, 545–555.

Farache, J., Koren, I., Milo, I., Gurevich, I., Kim, K.W., Zigmond, E., Furtado, G.C., Lira, S.A., and Shakhar, G. (2013). Luminal bacteria recruit CD103+ dendritic cells into the intestinal epithelium to sample bacterial antigens for presentation. Immunity *38*, 581–595.

Faure-André, G., Vargas, P., Yuseff, M.I., Heuzé, M., Diaz, J., Lankar, D., Steri, V., Manry, J., Hugues, S., Vascotto, F., et al. (2008). Regulation of dendritic cell migration by CD74, the MHC class II-associated invariant chain. Science *322*, 1705–1710.

Fenton, T.M., Wulff, L., Jones, G.-R., Vandamme, J., Jørgensen, B., Bain, C.C., Lee, J., Izarzugaza, J.M., Belling, K.G., Ho, T., and Nielsen, O.H. (2021). Single-cell characterisation of mononuclear phagocytes in the human intestinal 1 mucosa. bioRxiv. https://doi.org/10.1101/2021.03.28.437379.

Goswami, D., März, S., Li, Y.-T., Artz, A., Schäfer, K., Seelige, R., Pacheco-Blanco, M., Jing, D., Bixel, M.G., Araki, M., et al. (2017). Endothelial CD99 supports arrest of mouse neutrophils in venules and binds to neutrophil PILRs. Blood *129*, 1811–1822.

Goudot, C., Coillard, A., Villani, A.C., Gueguen, P., Cros, A., Sarkizova, S., Tang-Huau, T.-L., Bohec, M., Baulande, S., Hacohen, N., et al. (2017). Aryl hydrocarbon receptor controls monocyte differentiation into dendritic cells versus macrophages. Immunity *47*, 582–596, e6.

Hadis, U., Wahl, B., Schulz, O., Hardtke-Wolenski, M., Schippers, A., Wagner, N., Müller, W., Sparwasser, T., Förster, R., and Pabst, O. (2011). Intestinal tolerance requires gut homing and expansion of FoxP3+ regulatory T cells in the lamina propria. Immunity *34*, 237–246.

Hall, J.A., Cannons, J.L., Grainger, J.R., Dos Santos, L.M., Hand, T.W., Naik, S., Wohlfert, E.A., Chou, D.B., Oldenhove, G., Robinson, M., et al. (2011). Essential role for retinoic acid in the promotion of CD4+ T cell effector responses via retinoic acid receptor alpha. Immunity *34*, 435–447.

Heidkamp, G.F., Sander, J., Lehmann, C.H.K., Heger, L., Eissing, N., Baranska, A., Lühr, J.J., Hoffmann, A., Reimer, K.C., Lux, A., et al. (2016). Human lymphoid organ dendritic cell identity is predominantly dictated by ontogeny, not tissue microenvironment. Sci. Immunol. *1*, eaai7677.

Randolph, G.J., Beaulieu, S., Lebecque, S., Steinman, R.M., and Muller, W.A. (1998). Differentiation of monocytes into dendritic cells in a model of transendothelial trafficking. Science *282*, 480–483.

Hubo, M., Trinschek, B., Kryczanowsky, F., Tuettenberg, A., Steinbrink, K., and Jonuleit, H. (2013). Costimulatory molecules on immunogenic versus tolerogenic human dendritic cells. Front. Immunol. *4*, 82.

Iliev, I.D., Mileti, E., Matteoli, G., Chieppa, M., and Rescigno, M. (2009a). Intestinal epithelial cells promote colitis-protective regulatory T-cell differentiation through dendritic cell conditioning. Mucosal Immunol *2*, 340–350.

Iliev, I.D., Spadoni, I., Mileti, E., Matteoli, G., Sonzogni, A., Sampietro, G.M., Foschi, D., Caprioli, F., Viale, G., and Rescigno, M. (2009b). Human intestinal

epithelial cells promote the differentiation of tolerogenic dendritic cells. Gut *58*, 1481–1489.

Jacobelli, J., Friedman, R.S., Conti, M.A., Lennon-Dumenil, A.M., Piel, M., Sorensen, C.M., Adelstein, R.S., and Krummel, M.F. (2010). Confinement-optimized three-dimensional T cell amoeboid motility is modulated via myosin IIA-regulated adhesions. Nat. Immunol. *11*, 953–961.

Kabashima, K., Banks, T.A., Ansel, K.M., Lu, T.T., Ware, C.F., and Cyster, J.G. (2005). Intrinsic lymphotoxin-β receptor requirement for homeostasis of lymphoid tissue dendritic cells. Immunity *22*, 439–450.

Klebanoff, C.A., Spencer, S.P., Torabi-Parizi, P., Grainger, J.R., Roychoudhuri, R., Ji, Y., Sukumar, M., Muranski, P., Scott, C.D., Hall, J.A., et al. (2013). Retinoic acid controls the homeostasis of pre-cDC-derived splenic and intestinal dendritic cells. J. Exp. Med. *210*, 1961–1976.

Kuleshov, M.V., Jones, M.R., Rouillard, A.D., Fernandez, N.F., Duan, Q., Wang, Z., Koplev, S., Jenkins, S.L., Jagodnik, K.M., Lachmann, A., et al. (2016). Enrichr: a comprehensive gene set enrichment analysis web server 2016 update. Nucleic Acids Res *44*, W90–W97.

La Manno, G., Soldatov, R., Zeisel, A., Braun, E., Hochgerner, H., Petukhov, V., Lidschreiber, K., Kastriti, M.E., Lönnerberg, P., Furlan, A., et al. (2018). RNA velocity of single cells. Nature *560*, 494–498.

Lämmermann, T., Bader, B.L., Monkley, S.J., Worbs, T., Wedlich-Söldner, R., Hirsch, K., Keller, M., Förster, R., Critchley, D.R., Fässler, R., and Sixt, M. (2008). Rapid leukocyte migration by integrin-independent flowing and squeezing. Nature *453*, 51–55.

Lewis, K.L., Caton, M.L., Bogunovic, M., Greter, M., Grajkowska, L.T., Ng, D., Klinakis, A., Charo, I.F., Jung, S., Gommerman, J.L., et al. (2011). Notch2 receptor signaling controls functional differentiation of dendritic cells in the spleen and intestine. Immunity *35*, 780–791.

Liu, K., Waskow, C., Liu, X., Yao, K., Hoh, J., and Nussenzweig, M. (2007). Origin of dendritic cells in peripheral lymphoid organs of mice. Nat. Immunol. *8*, 578–583.

Luda, K.M., Joeris, T., Persson, E.K., Rivollier, A., Demiri, M., Sitnik, K.M., Pool, L., Holm, J.B., Melo-Gonzalez, F., Richter, L., et al. (2016). IRF8 transcription-factor-dependent classical dendritic cells are essential for intestinal T cell homeostasis. Immunity *44*, 860–874.

Mazzini, E., Massimiliano, L., Penna, G., and Rescigno, M. (2014). Oral tolerance can be established via gap junction transfer of fed antigens from CX3CR1+ macrophages to CD103+ dendritic cells. Immunity *40*, 248–261.

Muzumdar, M.D., Tasic, B., Miyamichi, K., Li, L., and Luo, L. (2007). A global double-fluorescent Cre reporter mouse. Genesis *45*, 593–605.

Nakano, H., Burgents, J.E., Nakano, K., Whitehead, G.S., Cheong, C., Bortner, C.D., and Cook, D.N. (2013). Migratory properties of pulmonary dendritic cells are determined by their developmental lineage. Mucosal Immunol *6*, 678–691.

Persson, E.K., Uronen-Hansson, H., Semmrich, M., Rivollier, A., Hägerbrand, K., Marsal, J., Gudjonsson, S., Håkansson, U., Reizis, B., Kotarsky, K., and Agace, W.W. (2013). IRF4 transcription-factor-dependent CD103+CD11b+ dendritic cells drive mucosal T helper 17 cell differentiation. Immunity *38*, 958–969.

Qiu, X., Hill, A., Packer, J., Lin, D., Ma, Y.A., and Trapnell, C. (2017). Single-cell mRNA quantification and differential analysis with census. Nat. Methods *14*, 309–315.

Sato, T., Vries, R.G., Snippert, H.J., van de Wetering, M., Barker, N., Stange, D.E., van Es, J.H., Abo, A., Kujala, P., Peters, P.J., and Clevers, H. (2009). Single Lgr5 stem cells build crypt-villus structures in vitro without a mesenchymal niche. Nature *459*, 262–265.

Schlitzer, A., McGovern, N., Teo, P., Zelante, T., Atarashi, K., Low, D., Ho, A.W.S., See, P., Shin, A., Wasan, P.S., et al. (2013). IRF4 transcription factor-dependent CD11b+ dendritic cells in human and mouse control mucosal IL-17 cytokine responses. Immunity *38*, 970–983.

Schlitzer, A., Sivakamasundari, V., Chen, J., Sumatoh, H.R.B., Schreuder, J., Lum, J., Malleret, B., Zhang, S., Larbi, A., Zolezzi, F., et al. (2015). Identification of cDC1- and cDC2-committed DC progenitors reveals early lineage priming at the common DC progenitor stage in the bone marrow. Nat. Immunol. *16*, 718–728.

Schneider, C.A., Rasband, W.S., and Eliceiri, K.W. (2012). NIH Image to ImageJ: 25 years of image analysis. Nat. Methods *9*, 671–675.

Schraml, B.U., Van Blijswijk, J., Zelenay, S., Whitney, P.G., Filby, A., Acton, S.E., Rogers, N.C., Moncaut, N., Carvajal, J.J., and Reis e Sousa, C. (2013). Genetic tracing via DNGR-1 expression history defines dendritic cells as a hematopoietic lineage. Cell *154*, 843–858.

Scott, C.L., Bain, C.C., Wright, P.B., Sichien, D., Kotarsky, K., Persson, E.K., Luda, K., Guilliams, M., Lambrecht, B.N., Agace, W.W., et al. (2015). CCR2(+) CD103(−) intestinal dendritic cells develop from DC-committed precursors and induce interleukin-17 production by T cells. Mucosal Immunol *8*, 327–339.

Shan, M., Gentile, M., Yeiser, J.R., Walland, A.C., Bornstein, V.U., Chen, K., He, B., Cassis, L., Bigas, A., Cols, M., et al. (2013). Mucus enhances gut homeostasis and oral tolerance by delivering immunoregulatory signals. Science *342*, 447–453.

Stuart, T., Butler, A., Hoffman, P., Hafemeister, C., Papalexi, E., Mauck, W.M., Hao, Y., Stoeckius, M., Smibert, P., and Satija, R. (2019). Comprehensive integration of single-cell data. Cell *177*, 1888–1902, e21.

Sujino, T., London, M., Hoytema van Konijnenburg, D.P.H., Rendon, T., Buch, T., Silva, H.M., Lafaille, J.J., Reis, B.S., and Mucida, D. (2016). Tissue adaptation of regulatory and intraepithelial CD4+ T cells controls gut inflammation. Science *352*, 1581–1586.

Sun, Y., Caplazi, P., Zhang, J., Mazloom, A., Kummerfeld, S., Quinones, G., Senger, K., Lesch, J., Peng, I., Sebrell, A., et al. (2014). PILRα negatively regulates mouse inflammatory arthritis. J. Immunol. *193*, 860–870.

Trapnell, C., Cacchiarelli, D., Grimsby, J., Pokharel, P., Li, S., Morse, M., Lennon, N.J., Livak, K.J., Mikkelsen, T.S., and Rinn, J.L. (2014). The dynamics and regulators of cell fate decisions are revealed by pseudotemporal ordering of single cells. Nat. Biotechnol. *32*, 381–386.

Van Der Maaten, L., and Hinton, G. (2008). Visualizing data using t-SNE. J. Mach. Learn. Res.

Vargas, P., Maiuri, P., Bretou, M., Saéz, P.J., Pierobon, P., Maurin, M., Chabaud, M., Lankar, D., Obino, D., Terriac, E., et al. (2016). Innate control of actin nucleation determines two distinct migration behaviours in dendritic cells. Nat. Cell Biol. *18*, 43–53.

Villablanca, E.J., Wang, S., de Calisto, J., Gomes, D.C.O., Kane, M.A., Napoli, J.L., Blaner, W.S., Kagechika, H., Blomhoff, R., Rosemblatt, M., et al. (2011). MyD88 and retinoic acid signaling pathways interact to modulate gastrointestinal activities of dendritic cells. Gastroenterology *141*, 176–185.

Yashiro, T., Yamaguchi, M., Watanuki, Y., Kasakura, K., and Nishiyama, C. (2018). The transcription factors PU.1 and IRF4 determine dendritic cell–specific expression of RALDH2. J. Immunol. *201*, 3677–3682.

Zen, K., Liu, Y., McCall, I.C., Wu, T., Lee, W., Babbin, B.A., Nusrat, A., and Parkos, C.A. (2005). Neutrophil migration across tight junctions is mediated by adhesive interactions between epithelial coxsackie and adenovirus receptor and a junctional adhesion molecule-like protein on neutrophils. Mol. Biol. Cell *16*, 2694–2703.

Zhang, Y., Conti, M.A., Malide, D., Dong, F., Wang, A., Shmist, Y.A., Liu, C., Zerfas, P., Daniels, M.P., Chan, C.C., et al. (2012). Mouse models of MYH9-related disease: mutations in nonmuscle myosin II-A. Blood *119*, 238–250.

Zheng, D., and Tian, B. (2017). Polyadenylation site-based analysis of transcript expression by 3′READS. Methods Mol. Biol. *1648*, 65–77.

## STAR★METHODS

### KEY RESOURCES TABLE

| REAGENT or RESOURCE | SOURCE | IDENTIFIER |
|---|---|---|
| Antibodies | | |
| Armenian Hamster IgG monoclonal anti-CD3 epsilon, APC/Cyanine7 conjugated, clone 145-2C11, dilution for flow cytometry – 1 to 200 | BioLegend | Cat#100330; RRID: AB_1877170 |
| Rat IgG2a kappa monoclonal anti-CD19, APC/Cyanine7 conjugated, clone 6D5, dilution for flow cytometry – 1 to 200 | BioLegend | Cat#115530; RRID: AB_830707 |
| Rat IgG2b kappa monoclonal anti-CD45, PE/Cyanine5.5 conjugated, clone 30-F11, dilution for flow cytometry – 1 to 500 | eBiosciences™ | Cat#35-0451-82; RRID: AB_469718 |
| Rat IgG2b kappa monoclonal anti- mouse CD16/CD32, Mouse BD Fc Block™, dilution for flow cytometry – 1 to 200 | BD Biosciences | Cat#553142; RRID: AB_394657 |
| Rat IgG2b kappa monoclonal anti-CD11b, PE/Cyanine7 conjugated, clone M1/70, dilution for flow cytometry – 1 to 200 | eBiosciences™ | Cat#25-0112-82; RRID: AB_469588 |
| Rat IgG2b kappa monoclonal anti- mouse I-A/I-E, Alexa Fluor™ 700 conjugated, clone M5/114.15.2, dilution for flow cytometry – 1 to 400 | BioLegend | Cat#107622; RRID: AB_493727 |
| Armenian Hamster IgG1 λ2 monoclonal anti-CD11c, APC conjugated, clone HL3, dilution for flow cytometry – 1 to 200 | BD Biosciences | Cat#550261; RRID: AB_398460 |
| Mouse IgG1 kappa monoclonal anti-CD64, Brilliant Violet™ 421 conjugated, clone X54-5/7.1, dilution for flow cytometry – 1 to 200 | BioLegend | Cat#139309; RRID: AB_2562694 |
| Armenian Hamster IgG monoclonal anti-CD103, PE conjugated, clone 2E7, dilution for flow cytometry – 1 to 200 | eBiosciences™ | Cat#12-1031-82; RRID: AB_465799 |
| Rat DA/HA IgG2b kappa monoclonal anti – CD11b, Brilliant Violet™ 421 conjugated, clone M1/70, dilution for cryosections – 1 to 100; dilution for whole-mount – 1 to 20 | BD Biosciences | Cat#562605; RRID: AB_11152949 |
| Goat IgG polyclonal anti-CD103, unconjugated, dilution for cryosections – 1 to 100; dilution for whole-mount – 1 to 50 | R&D systems | Cat#AF1990; RRID: AB_2128618 |
| Armenian Hamster IgG monoclonal anti-CD103, Alexa 488 conjugated, clone 2E7, dilution for flow cytometry – 1 to 200 | BioLegend | Cat#121408; RRID: AB_535950 |
| Rat IgG2a kappa monoclonal anti-CCR7, PE/Cy5 conjugated, clone 4B12, dilution for flow cytometry – 1 to 50 at 37°C | BioLegend | Cat#120114; RRID: AB_2072905 |
| Rat IgG1 kappa monoclonal anti-CD83, PE conjugated, clone Michel-19, dilution for flow cytometry – 1 to 200 | BioLegend | Cat#121508; RRID: AB_572015 |
| Mouse IgG2c monoclonal anti-CD209a, PE conjugated, clone MMD3, dilution for flow cytometry – 1 to 200 | BioLegend | Cat#833003; RRID: AB_2721636 |

(*Continued on next page*)

*Continued*

| REAGENT or RESOURCE | SOURCE | IDENTIFIER |
|---|---|---|
| Armenian Hamster IgG2 kappa monoclonal anti-CD80, PE conjugated, clone 16-10A1, dilution for flow cytometry – 1 to 100 | BD Biosciences | Cat#553769; RRID: AB_395039 |
| Rat IgG2a kappa monoclonal anti-CD86, BV605 conjugated, clone GL-1, dilution for flow cytometry – 1 to 200 | BioLegend | Cat#105037; RRID: AB_11204429 |
| Rat DA monoclonal anti-CD4, APC conjugated, clone RM4-5, dilution for flow cytometry – 1 to 100 | BD Biosciences | Cat#553051; RRID: AB_398528 |
| Armenian Hamster IgG monoclonal anti-CD69, eFluor450 conjugated, clone H1.2F3, dilution for flow cytometry – 1 to 300 | eBiosciences™ | Cat#48-0691-82; RRID: AB_10719430 |
| Mouse IgG1 kappa monoclonal anti-TCR, PE conjugated, clone MR9-4, dilution for flow cytometry – 1 to 300 | BD Biosciences | Cat#553190; RRID: AB_394698 |
| Rat IgG2a kappa monoclonal anti-a4b7, APC conjugated, clone DATK-32, dilution for flow cytometry – 1 to 100 | eBiosciences™ | Cat#17-5887-82; RRID: AB_1210577 |
| Rat IgG2a kappa monoclonal anti-B220, PE conjugated, clone RA3-6B2, dilution for flow cytometry – 1 to 200 | BD Biosciences | Cat#561878; RRID: AB_10893353 |
| Rat IgG1 kappa monoclonal anti-SIRPa, PerCP/eFluor 710 conjugated, clone P84, dilution for flow cytometry – 1 to 400 | eBiosciences™ | Cat#46-1721-82; RRID: AB_10804639 |
| Rat IgG2a kappa monoclonal anti-CD135, PE conjugated, clone A2F10, dilution for flow cytometry – 1 to 100 | BioLegend | Cat#135306; RRID: AB_1877217 |
| Armenian Hamster IgG monoclonal anti-CD11c, PE/Cy7 conjugated, clone N418, dilution for flow cytometry – 1 to 600 | BioLegend | Cat#117318; RRID: AB_493568 |
| Rat IgG2a kappa monoclonal anti-CD115, APC/Cy7 conjugated, clone AFS98, dilution for flow cytometry – 1 to 100 | BioLegend | Cat#135532; RRID: AB_2632740 |
| Rat IgG2b kappa monoclonal anti-SIGLEC H, eFluor450 conjugated, clone eBio440c, dilution for flow cytometry – 1 to 800 | eBiosciences™ | Cat#48-0333-82; RRID: AB_2574015 |
| Rat IgG2b kappa monoclonal anti-CD11b, BV605 conjugated, clone M1/70, dilution for flow cytometry – 1 to 1000 | BioLegend | Cat#101237; RRID: AB_11126744 |
| Armenian Hamster IgG monoclonal anti-CD3e, FITC conjugated, clone 145-2C11, dilution for flow cytometry – 1 to 800 | BioLegend | Cat#100306; RRID: AB_312671 |
| Rat IgG2a kappa monoclonal anti-NKP46, FITC conjugated, clone 29A1.4, dilution for flow cytometry – 1 to 300 | BioLegend | Cat#137606; RRID: AB_2298210 |
| Mouse IgA monoclonal anti-CD19, FITC conjugated, clone MB19-1, dilution for flow cytometry – 1 to 200 | BioLegend | Cat#101506; RRID: AB_312825 |
| Rat IgG2b kappa monoclonal anti-Ter119, FITC conjugated, clone TER-119, dilution for flow cytometry – 1 to 100 | BioLegend | Cat#116206; RRID: AB_313707 |
| Rat IgG2a kappa monoclonal anti-Ly-6G, FITC conjugated, clone 1A8, dilution for flow cytometry – 1 to 1000 | BioLegend | Cat#127606; RRID: AB_1236494 |

(*Continued on next page*)

*Continued*

| REAGENT or RESOURCE | SOURCE | IDENTIFIER |
| --- | --- | --- |
| Rat IgG2c kappa monoclonal anti-Ly-6C, APC conjugated, clone HK4.1, dilution for flow cytometry – 1 to 1000 | BioLegend | Cat#128016; RRID: AB_1732076 |
| Rabbit IgG polyclonal anti-Laminin, unconjugated, dilution for cryosections – 1 to 200, dilution for whole-mount – 1 to 100 | Sigma-Aldrich | Cat#L9393; RRID: AB_477163 |
| Ultra-LEAF Purified anti-mouse CD275 (B7-H2, B7-RP1, ICOS Ligand), clone HK5.3 | BioLegend | Cat#107410; RRID: AB_11149485 |
| Ultra-LEAF Purified Rat IgG2a, kappa isotype Ctrl, clone RTK2758 | BioLegend | Cat#400543; RRID: AB_11148951 |
| Ultra-LEAF Purified anti human/mouse TGF-b1, clone 19D8 | BioLegend | Cat#521707; RRID: AB_2810653 |
| Rabbit monoclonal anti RARA, clone SN0725, dilution for flow cytometry – 1 to 100 | Invitrogen™ | Cat#MA5-32325; RRID: AB_2809606 |
| Rat IgG1 kappa monoclonal anti-TNFa, PE conjugated, clone MP6-XT22, dilution for flow cytometry – 1 to 100 | BioLegend | Cat#506305; RRID: AB_315426 |
| Rabbit monoclonal anti-IL-1b, unconjugated, dilution for flow cytometry – 1 to 100 | Cell Signaling | Cat#12703S; RRID: AB_2737350 |
| Mouse IgG2a kappa monoclonal anti-CD45.1, BV605 conjugated, clone A20, dilution for flow cytometry – 1 to 200 | BioLegend | Cat#110738; RRID: AB_2562565 |
| Mouse IgG2a kappa monoclonal anti-CD45.2, PerCP/Cy5.5 conjugated, clone 104, dilution for flow cytometry – 1 to 100 | BD Biosciences | Cat#552950; RRID: AB_394528 |
| Armenian Hamster IgG monoclonal anti-CD103, PerCP/eFluor 710 conjugated, clone 2E7, dilution for flow cytometry – 1 to 200 | eBiosciences™ | Cat#46-1031-82; RRID: AB_2573704 |
| Rat IgG2b kappa monoclonal anti-CD117, PE/Cy5 conjugated, clone 2B8, dilution for flow cytometry – 1 to 200 | BioLegend | Cat#105810; RRID: AB_313219 |
| Rat IgG2a kappa monoclonal anti-ICOS-l, PE conjugated, clone HK5.3, dilution for flow cytometry – 1 to 200 | BioLegend | Cat#107405; RRID: AB_2248797 |
| Mouse IgG1 kappa monoclonal anti-CD64, APC conjugated, clone X54-5/7.1, dilution for flow cytometry – 1 to 200 | BioLegend | Cat#139306; RRID: AB_11219391 |
| Chemicals, peptides, and recombinant proteins | | |
| WIN18446 (Bisdiamine) | Tocris | Cat#4736 |
| CellTrace™ CFSE Cell proliferation Kit | Invitrogen™ | Cat#C34554 |
| CellTrace™ Violet Cell proliferation Kit | Invitrogen™ | Cat#C34557 |
| LIVE/DEAD™ Fixable Aqua Dead Cell Stain Kit, dilution for flow cytometry – 1 to 1000 in protein-free buffer | Thermo Fischer Scientific | Cat#L34965 |
| Fixable Viability Dye eFluor™ 780, dilution for flow cytometry – 1 to 2000 | eBiosciences™ | Cat#65-0865-14 |
| LIVE/DEAD™ Fixable Red Dead Cell Stain Kit, dilution for flow cytometry – 1 to 1000 | Thermo Fischer Scientific | Cat#L34972 |
| Ovalbumin, Low endo, Purified | Worthington Biochemical Corporation | Cat#LS003062 |

(*Continued on next page*)

| *Continued* | | |
|---|---|---|
| REAGENT or RESOURCE | SOURCE | IDENTIFIER |
| Peptide OVA 323-339 | Invivogen | Cat#vac-isq |
| Ovalbumin, Texas RedTM Conjugate | Thermo Fischer Scientific | Cat#O23021 |
| Recombinant Murine Noggin | Peprotech | Cat#250-38 |
| Recombinant Mouse R-spondin 1 | R&D systems | Cat#3474-RS-050 |
| Recombinant Murine EGF | Peprotech | Cat#315-09 |
| Recombinant Murine FGF | Peprotech | Cat#450-33 |
| MUC2 | Sigma Aldrich | Cat# M2378 |
| Critical commercial assays | | |
| EdU FC Kit 488 | baseclick | Cat#BCK488-IV-FC-S |
| Aldefluor$^{TM}$ Kit | Stemcell Technologies | Cat#01700 |
| BD Cytofix/CytopermTM Solution Kit | BD Biosciences | Cat#554714 |
| Deposited data | | |
| Single Cell RNaseq datasets | This paper | Gene Expression Omnibus (GEO): GSE188379 |
| Experimental models: Organisms/strains | | |
| Mouse: C57BL/6J | Charles River | JAX:000664 |
| Mouse: mT/mG | Lequn Luo (Stanford) | Muzumdar et al., 2007 |
| Mouse: CD11c-Cre | S. Amigorena (Institut Curie) | Caton et al., 2007 |
| Mouse: MyoIIGFP | AM. Lennon (Institut Curie) | Zhang et al., 2012 |
| Mouse: PiIralpha KO | A. Zarrin (Genentech Inc) | Sun et al., 2014 |
| Mouse: *Clec9a*$^{+/cre}$Rosa$^{+/EYFP}$ | C. Reis e Sousa (Francis Crick Institut) | Schraml et al., 2013 |
| Mouse: Flt3l KO | P. Guermonprez (Université de Paris) | JAX:37395-JAX |
| Mouse: CD11c-YFP | AM. Lennon (Institut Curie) | JAX:008829 |
| Mouse: OT-II RAG2-KO THY1.1 BL/6N | O. Lantz (Institut Curie) | N/A |
| Mouse: CD45.1 BL/6N | S. Amigorena (Institut Curie) | JAX:002014 |
| Mouse: CCR7-GFP KO/KI | The Jackson Laboratory | JAX:027913 |
| Mouse: CCR2 KO | E. Gautier (Hôpital de la Pitié-Salpétrière) | JAX:004999 |
| Mouse: MyoIIA flox/flox | AM. Lennon (Institut Curie) | Jacobelli et al., 2010 |
| Mouse: B6J;B6N-Tyrc-Brd Arpc4tm1a(EUCOMM)Wtsi/WtsiOulu | Wellcome Trust Sanger Institute | MGI:4433308 |
| Software and algorithms | | |
| FlowJo v10 | https://www.flowjo.com/ | https://www.flowjo.com/ |
| GraphPad Prism v8 | https://www.graphpad.com/scientific-software/prism/ | https://www.graphpad.com/scientific-software/prism/ |
| Image J | Schneider et al., 2012 | https://imagej.nih.gov/ij/ |
| Enrichr | Chen et al., 2013; Kuleshov et al., 2016 | http://amp.pharm.mssm.edu/Enrichr/ |

## RESOURCE AVAILABILITY

### Lead contact

Further information and requests for resources and reagents should be directed to and will be fulfilled by the lead contact, Ana-Maria Lennon-Duménil (amlennon@curie.fr).

CellPress
OPEN ACCESS

## Materials availability

This study did not generate new unique reagents.

## Data and code availability

The scRNAseq datasets generated in this study are available for download at the Gene Expression Omnibus (GEO): GSE188379.

This paper does not report original code.

Any additional information required to reanalyze the data reported in this paper is available from the lead contact upon request.

# EXPERIMENTAL MODEL AND SUBJETC DETAILS

## Mice

Conditional MyoIIA deficient mice were generated by crossing *Myh9*$^{flox/flox}$ mice (Jacobelli et al., 2010) with *Itgax*$^{Cre+/-}$ mice (Caton et al., 2007). B6J;B6N-Tyrc-Brd Arpc4tm1a(EUCOMM)Wtsi/WtsiOulu mice generated by the European Conditional Mouse Mutagenesis Program (EUCOMM) were obtained from the Wellcome Trust Sanger Institute. Then, backcrossed with Flp recombinase transgenic mice and C57BL/6 to create conditional *Arpc4* $^{flox/flox}$ mice. *Arpc4* $^{flox/flox}$ X *Itgax*$^{Cre+/-}$ mice were generated by crossing *Arpc4*$^{flox/flox}$ mice with *Itgax*$^{Cre}$ mice, bred and maintained in our animal facility (Institut Curie, Paris, France) until use. *Pilra*$^{-/-}$ mice (Sun et al., 2014) were kindly provided by Genentech,Inc. (MTA OM-217467), imported and maintained in our animal facility. For *Myh9* $^{flox/flox}$ X *Itgax*$^{Cre+/-}$, *Arpc4* $^{flox/flox}$ X *Itgax*$^{Cre+/}$, and *Pilra*$^{-/-}$ mice, littermates were used for the analyses. *Clec9a*$^{+/cre}$Rosa$^{+/EYFP}$ mice (Schraml et al., 2013) were bred and maintained in the Francis Crick Institute, London animal facility. Green fluorescent protein (GFP)-Myosin IIA heavy chain mice previously described (Zhang et al., 2012), *Itgax*: Cre/R26$^{mTmG}$ mice previously described for CD11c compartment labeling (Chikina et al., 2020), OT-II *Rag2*$^{-/-}$ THY1.1 BL/6N, CD45.1 BL/6N and *Itgax*-EYFP mice were bred and maintained in our animal facility. C57BL/6J CD45.2 mice were purchased from Charles River and maintained in our animal facility until use. *Flt3l*$^{-/-}$ mice were bred in the animal facility of Xavier Bichat Faculty of Medicine (Paris Diderot University, France) and kindly provided by Pierre Guermonprez. *Ccr2*$^{-/-}$ mice were kindly provided by Emmanuel Gautier. *Ccr7*$^{gfp}$ reporter mouse (C57BL/6-*Ccr7*$^{tm1.1Dnc}$/J, JAX stock #027913) (Nakano et al., 2013) were originally purchased from The Jackson Laboratory and bred in our mice facility after. Vitamin A deficient or excess diet were purchased from Ssniff (Soest, Germany) and mice were fed for 3 months. Experiments were performed on 8 to 14 weeks-old male or female mice. For animal care, we strictly followed the European and French National Regulation for the Protection of Vertebrate Animals used for Experimental and other Scientific Purposes (Directive 2010/63; French Decree 2013-118, Authorization APAFIS#28256-2020081317392135 v2 given by National Authority), and protocols approved by the Stockholm Regional Ethics Committee.

# METHODS DETAILS

## Isolation of Intestinal Cell Suspensions

For the preparation of single-intestinal-cell suspension the small intestines were extracted from mice by separation from the mesentery. Peyer patches were removed, and intestines were opened with scissors along the intestinal length, then washed in PBS. Next, intestinal tissues were incubated on a magnetic shaker in a complete medium (CM, 2% FBS in $Ca^{2+}$, $Mg^{2+}$-free Phenol Red 1X HBSS; H4385 Sigma-Aldrich, St. Louis, MO, USA diluted in filtered $H_2O$) in the presence of 1 mM DTT (D9779, Sigma-Aldrich, St. Louis, MO, USA), 5mM EDTA (15575-038 from Invitrogen) and 15mM HEPES (15630-056 from Gibco) at 37°C for 30 min to recover epithelial fraction, and subsequently incubated with 1 mM EDTA in 5% FBS/ PBS at 37°C for 10 min. This was followed by incubation with 15mM Hepes in 1% FBS/PBS at room temperature for 7min without agitation. The supernatants containing intestinal epithelial fraction were collected and analyzed by flow cytometry. Isolated tissues were further digested using 0.15 mg/mL Liberase (054010200001 from Roche) and 0.1mg/mL DNAse1 (10104159001 from Roche) in HBSS at 37°C for 45min with magnetic agitation. Tissues were then homogenized, filtered on 100μm cell strainer, and washed in HBSS. Gradient was performed on both epithelial fraction and lamina propria in 44% and 67% fractions of Percoll (17-0891-01, GE Healthcare) prepared in 10mM Hepes in HBSS. Single-cell suspensions were stained with mouse antibodies and analyzed by flow cytometry.

## Flow cytometry analysis

Cells were stained in 2mM EDTA, 5%FBS in PBS. RALDH activity in individual cells was measured using an Aldefluor kit according to the manufacturer's protocol. 7.5 μM of DEAB was added in different tubes at 37°C for 15 min as Aldefluor fluorescent baseline control. Intracellular staining of cytokines was performed with BD Cytofix/Cytoperm™ Solution Kit, after obtention of cells and incubation with PMA (10ng/ml), Ionomycin (1μg/ml) and Brefeldin A (1μg/ml), during 4h at 37°C/ 5% $CO_2$. Flow cytometry was performed on Fortessa (BD), LSR II (BD) and FACSAria (BD), and analyzed using FlowJo software version 10. Percentage values were charted with Graphpad Prism version 8.

## Single-cell RNA sequencing

### *Single-cell RNAseq library preparation and sequencing*

CD103$^{+}$CD11b$^{+}$ dendritic cells were sorted from the lamina propria and epithelium of 2 pooled small intestines of C57BL/6J mice. Cellular suspensions were loaded on a 10X Chromium instrument (10X Genomics) according to the manufacturer's protocol based

on the 10X GEMCode proprietary technology. Single-cell RNA-Seq libraries were prepared using Chromium Single Cell 3' v2 Reagent Kit (10X Genomics) according to manufacturer's protocol as described in Goudot et al., 2017. The sequencing was performed using a Rapid Run flowcell of HiSeq 2500 (Illumina) in Paired-end 26/98 to target 100M reads per sample. With an average of 1000 cells per sample, the obtained coverage corresponds to 100,000 reads/cell.

#### Single-cell RNAseq data processing and analysis

Cell Ranger (version 2.0.1) (from 10x genomics) was used to process Chromium single cell 3' v2 RNA-seq output files. First, *cellranger mkfastq* (with default parameter) was applied to generate fastq files for the Read1 for cell barcode and UMI and Read2 for transcript. After, *cellranger count* (with default parameters) aligned the Read2 to the mouse reference genome mm10 using STAR (version 2.5.1) (Dobin et al., 2013). Further analysis was performed using Seurat package (version 2.3.4) in R (version 3.4.0) (Stuart et al., 2019). For the filtering step, we excluded the poor-quality cells such as cells with less than 200 unique genes and more than 3600 unique genes per cell (as they are potentially cells doublets). Only genes expressed in 3 or more cells have been preserved. Finally, after filtering step about 1% of cells were discarded and for the rest of the analysis, we used an expression matrix resulting in 13316 genes across 2318 cells (among 2341 cells). The matrix was normalized using gene expression values for each cell, was divided by the total number of transcripts and multiplied by 10,000. Then, these values were natural log-transformed before downstream analysis.

For dimensionality reduction analysis, we first identified 2634 genes as highly variable genes across the single cells (cutoff value for dispersion = 0.5; cutoff value for average expression = 0). Then we performed PCA using the variable genes as input and determined 20 PCs as significant PCs. These principal components were used as input for t-Distributed Stochastic Neighbor Embedding (tSNE) (Van Der Maaten and Hinton, 2008). Clusters were identified using the shared nearest neighbor (SNN) modularity optimization based clustering algorithm from the Seurat package (*FindClusters* function with the following parameters: dims.use = 1:20, resolution = c(seq(0, 1.5, by = 0.1)), n.iter = 10000, force.recalc = T; all other parameters are default settings). Clustree analysis (clustree R package, version 0.2.2) was used by changing the resolution parameters from 0 to 1.5. Finally, we kept a resolution parameter at 1.0 and we defined nine clusters. After controlling the expression of some quality control genes, we excluded the clusters with cells expressing Cd8a or Mafb and cells that do not express Itgam. These clusters of cells were considered as contaminating cells, then we finally analyzed five clusters.

Cell specific marker genes were identified by comparing cells in a specific cluster with cells in all other clusters using *FindAllMarkers* from Seurat package (wilcoxon test; logFC threshold = 0.25; only positive markers). Heatmap, feature plots and violins plot were performed using Seurat package.

To construct single cell pseudotime trajectory we applied Monocle2 (version 2.6.4) (Qiu et al., 2017; Trapnell et al., 2014) using 3540 differential expressed genes using *differentialGeneTest* from Monocle2 (q value < 0.01). Cells were ordered along the inferred trajectory to indicate their differentiation progress. Then, the pseudotime trajectory was visualized on the reduced dimensional space.

Pathway analyses were performed using Enrichr (Chen et al., 2013; Kuleshov et al., 2016).

RNA velocity analysis was performed by GenoSplice technology (www.genosplice.com) using RNA Velocity (La Manno et al., 2018). Spliced and unspliced expression matrices were generated using the standard velocyto pipeline for Epi and LP samples. Loom files were merged using loompy package on Python. R packages velocyto.R and SeuratWrappers were then used to estimate RNA velocity vectors with velocity parameters kCells = 25, fit.quantile = 0.2 and deltaT = 1 and visualization parameters n = 200, grid.n = 40, arrow.scale = 3 and scale = "sqrt."

### Tissue immunofluorescence

The small intestine was extracted and washed by flushing the lumen with cold Leibovitz's L-15 medium (L5520, SIGMA). 5mm fragments from the small intestine were obtained and tissue was fixed in the fixative solution (4% PFA, 0.05 M L-Lysine, 12mM $NaH_2PO_4$, 50mM $Na_2HPO_4$ in $H_2O$) at 4°C overnight, and dehydrated in 20% sucrose in PBS for 4h at room temperature. After washing twice with 40 mM $NaH_2PO_4$, 160 mM $Na_2HPO_4$ in $H_2O$, samples were embedded with OCT in tissue cassettes, snap frozen using liquid nitrogen, and stored at -80°C. Samples were incubated in permeabilization buffer (1% Triton X100 in PBS) for 1h, then in blocking buffer (1% BSA,3% FCS,0,2% Triton-X100 in PBS) for 1h. Tissue staining was performed by incubating gut slices with primary antibodies overnight in 0,2% Tx100 in PBS (100μL/3slices), using the following dilutions: 1/50 for anti-CD103; 1/100 for all other antibodies diluted. Samples were washed 3 times 0,2%Tx100 in PBS for 1h (each wash), with mild shaking rocking. When required, samples were incubated with secondary antibodies overnight (both diluted 1/100) and then washed as described before. Samples were kept at room temperature during all steps before mounting with Aqua Polymount medium. Z-stacks consisting of 1024*1024 pixels (150nm pixel size) images spaced by 0.35μm were acquired using an inverted confocal microscope (Leica DMi8, SP8 scanning head unit) equipped with a 63X oil immersion objective, pixel size 1024*1024, z-step 0,35mm.

### Quantitative real-time RT-PCR

$CD103^+CD11b^+$ dendritic cells were purified from small intestine LP or epithelium and pooled from 4 C57BL/6J mice per experiment. RNA was obtained using the RNeasy Plus Micro Kit (Qiagen) and RT-qPCR was performed with SYBR Green Master mix after single-stranded cDNA obtention using the high capacity cDNA synthesis kit (Thermo Fisher) according to manufacturer's protocol. Oligonucleotides primers were synthesized by Eurogentec. Defa24 primers were designed as from Castillo et al., 2019. Housekeeping Hypoxanthine guanine phosphoribosyl transferase (Hprt) gene primers were designed using Primer-BLAST: Fw 5' CAGTCCCA GCGTCGTGATTA 3', Rv 5' TGGCCTCCCATCTCCTTCAT 3'. Products obtained after normal PCR reaction with the designed primers were run in an agarose gel, to corroborate the unique product amplification and correct amplicon size. Quantitative PCR was

CellPress
OPEN ACCESS

performed in a Lyght Cycler 480 termocycler (Roche). Data were normalized to Hprt and to values obtained in LP cDC2s that were used as a base unit equal to one, then fold change of intraepithelial over LP cDC2s was displayed, calculated by the formula 2^-ΔΔCT.

## Adoptive transfer of preDCs

PreDC transfer experiments were performed as previously described (Scott et al., 2015). Briefly, 2 x $10^6$ B16 Flt3l-producing tumor cells were injected subcutaneously in WT CD45.2 mice and after 15-20 days, preDCs were sorted from BM. 6.5 x $10^5$ CellTrace Violet proliferation dye (eBioscience) labeled cells were injected intravenously into CD45.1 recipient mice, and their differentiation dynamic was followed in small intestine after 4, 7 and 10 days post-transfer by flow cytometry. Total epithelium sample was acquired by flow cytometry for the small intestine epithelium, corresponding to around 3-3.5 million events per sample. For LP, 4 million of cells were acquired by sample, to ensure data robustness.

## EdU turnover rate analysis

C57BL/6J mice were injected I.P. with 25mg/kg of EdU (baseclick) and $EdU^+$ cells were detected after 1, 4 or 10 post single injection by flow cytometry following manufacturer's protocol.

## Immunoblotting

Immunoblotting was performed as previously described (Vargas et al., 2016). Briefly, DCs were lysed for 2 min in a buffer containing 100 mM Tris, 150 mM NaCl, 0.5% NP-40 and a protease inhibitor cocktail tablet (Roche). Fifty micrograms of soluble extracts were loaded onto a 4–20% TGX gradient gel (BioRad) and transferred onto a Trans-Blot Turbo PVDF/Nitrocellulose membrane (BioRad). The membrane was blocked, incubated sequentially with the appropriate antibodies and revealed using the SuperSignal West Dura substrate (Thermo Scientific).

## Antibiotic and anti-fungal treatment

C57BL/6 were gavaged during 10 days with 200μL per day of PBS or 0.5mg/mL Fluconazole (F8929) in PBS or antibiotic cocktail in PBS of Ampicillin A9393 1mg/mL+ Gentamicin sulfate G4918 1mg/mL+ Vancomycin 861987 0,5mg/mL+ Metronidazole M1547 1mg/mL+ Neomycin trisulfate salt N1876 1mg/mL (all products purchased at Sigma-Aldrich).

## Inhibition of RALDH activity

Mice were gavaged for 2 days with 230μL olive oil or 0,1mg/kg of Bisdiamine (WIN 18446, ref 1477-57-2 from Tocris) diluted in olive oil. RALDH activity was assessed by flow cytometry with the ALDEFLUOR kit (StemCell).

## Micro-channels preparation and analysis

Micro-channels were prepared as previously described (Faure-André et al., 2008; Vargas et al., 2016). Polydimethylsiloxane (PDMS) (GE Silicones) was used. Micro-channel surface was coated with 10 μg/ ml bovine plasma fibronectin (Sigma) for 1 h and then washed with PBS before introduction of cells in complete medium with or without ATRA (1nM). For visualization of nucleus, we add in the medium NucBlue Live Ready Probes Reagent (Hoechst 33342, Thermo Scientific #R37605). Migrating cells were imaged for 16 h on an epifluorescence video-microscope Nikon TiE microscope equipped with a cooled CCD (charge-coupled device) camera (HQ2, Photometrics) with a ×20 objective. A frequency of acquisition of 1 image per 1 min of transmission phase was used. Image processing and analysis was performed using ImageJ. Migrating cell kymographs were generated by subtracting from each frame the mean projection of the whole movie, generating clear objects in a dark background. This was after analyzed using a custom program as described in Faure-André et al., 2008 and with a custom ImageJ macro to analyze MyoIIA GFP intensity. For analysis each channel was divided in three zones according to constriction position.

## Transwell experiments

Sorted lamina propria $CD103^+CD11b^+$ DCs were added on the top part of 96 well 3μm-pore permeable supports Transwell® and let them transmigrate overnight at 37°C with 5% $CO_2$ in supplemented RPMI medium (10% FBS, Glutamine 1X, Pen/Strep 1X, 0.05nM B-mercaptoethanol). DCs were obtained from the upper and lower compartments and analyzed by flow cytometry.

## DC co-culture with epithelial cells and mucus

$CD103^+CD11b^+$ dendritic cells were sorted from small intestine lamina propria of 4 pool C57BL/6J mice and incubated with freshly obtained small intestinal epithelial cells in a ratio 1:10, with intestinal epithelial cell supernatant or alone, overnight at 37°C with 5% $CO_2$ in supplemented RPMI medium and analyzed by flow cytometry. ATRA (1nM), Bisdiamine (45 μM) or antiTGF-beta 10μg/ml were added at the moment of co-culture. For mucus experiments, sorted $CD103^+CD11b^+$ dendritic cells were incubated overnight in the presence or absence of 50 μg/ml porcine MUC2 (Sigma Aldrich) at 37°C with 5% $CO_2$, and analyzed by flow cytometry.

### Organoid-DC co-culture

Mouse intestinal crypts from duodenum were isolated as described in Sato et al., 2009. Briefly, cleaned duodenum was cut in very small pieces and incubated for 30 minutes at 4°C in PBS containing 2mM of EDTA. Crypts were obtained after three cycles of vigorous shaking and filtering through 70 µm cell strainers, and a final centrifugation at 100xg for 6 min. Organoids were maintained in Matrigel with ENR medium: DMEM F/12, antibiotic-antimycotic 2X, Glutamax 2,5X, B27 1X, N2 1X, Noggin 100ng/ml, EGF 20ng/ml, mbFGF 10ng/ml, R-spondin 1 500ng/ml. Media was changed after 2 days, and on day 3 after isolation, purified LP $CD103^+CD11b^+$ dendritic cells were added to the culture. ENR medium was changed by supplemented RPMI medium to ensure dendritic cells viability during co-culture. After overnight incubation, dendritic cells were obtained from the supernatant and from inside the Matrigel by disrupting the Matrigel by strong pipetting and incubation with Trypsin for 6min. Cells were after stained and analyzed by flow cytometry. For imaging, LP $CD103^+CD11b^+$ dendritic cells were sorted from CD11c-YFP reporter mice, co-cultured overnight with membrane-fluorescent (mTmG mice) derived organoids in 8 well ibidi chambers and imaged in live using an inverted confocal microscope (Leica DMi8, SP8 scanning head unit) equipped with a 40X oil immersion objective.

### Antigen presentation and capturing assay

$CD103^+CD11b^+$ dendritic cells were sorted from the lamina propria and epithelium of 4 pooled small intestines of C57BL/6J mice. For antigen presentation assay, after isolation, cells were plated in round bottom 96 well plates and pre-incubated with different concentrations of Ovalbumin: 2 mg/mL, 1mg/mL and 0.5 g/mL, or OVA peptide II at 10 µg/mL for 5 hours at 37°C with 5% $CO_2$. After extensive washing, DCs were incubated with CFSE-labeled OT-II T cells in a ratio of 1:10. OT-II T cell activation was analyzed 18 hours after, and after 3 days, proliferation was measured by flow cytometry. Supernatants were collected after 3 days and the concentration of IL2 was analyzed by Luminex. For antigen presentation assay with anti-ICOS-L antibodies, the antibody or isotype was added at the moment of OT-II – DC co-culture at 50 µg/ml. For re-activation of T cells, 1 day after DC-T cells co-culture, OT-II T cells were plated on anti CD3 coated 96 well plates, and anti CD28 antibody was added soluble. For antigen capture assay, after sorting, cells were incubated with fluorescent Ovalbumin Texas Red (0,1 mg/mL and 0,2 mg/mL) at 37°C with 5% CO2 for 1 hour, washed and analyzed by flow cytometry; negative control was incubated at 0°C during the same time.

### Cytospin and MGG staining

Purified gut $CD103^+CD11b^+$ dendritic cells were centrifuged at 700rpm for 5 minutes in the cytocentrifuge. Cells were stained with May-Grünwald dye for 3 minutes, washed with neutral water and stained for 20 minutes with Giemsa dye diluted at 15% in neutral water.

## QUANTIFICATION AND STATISTICAL ANALYSIS

Number of mice and experiments, and statistical tests are reported in each figure legend. Analyses were performed using GraphPad Prism 8 software. Statistical significance was calculated using t test (paired or unpaired) or Mann-Whitney, one-way ANOVA or Kruskal-Wallis test, or two-way ANOVA according to test requirements. Error bars represent SEM and p values <.05 were considered statistically significant (* $p$ <.05, ** $p$ <.01, *** $p$ <.001, **** p < ,0001).

Immunity, Volume *55*

# Supplemental information

# Epithelial colonization by gut dendritic cells promotes their functional diversification

**Claudia A. Rivera, Violaine Randrian, Wilfrid Richer, Yohan Gerber-Ferder, Maria-Graciela Delgado, Aleksandra S. Chikina, Annika Frede, Chiara Sorini, Mathieu Maurin, Hana Kammoun-Chaari, Sara M. Parigi, Christel Goudot, Mar Cabeza-Cabrerizo, Sylvain Baulande, Sonia Lameiras, Pierre Guermonprez, Caetano Reis e Sousa, Marc Lecuit, Hélène D. Moreau, Julie Helft, Danijela Matic Vignjevic, Eduardo J. Villablanca, and Ana-Maria Lennon-Duménil**

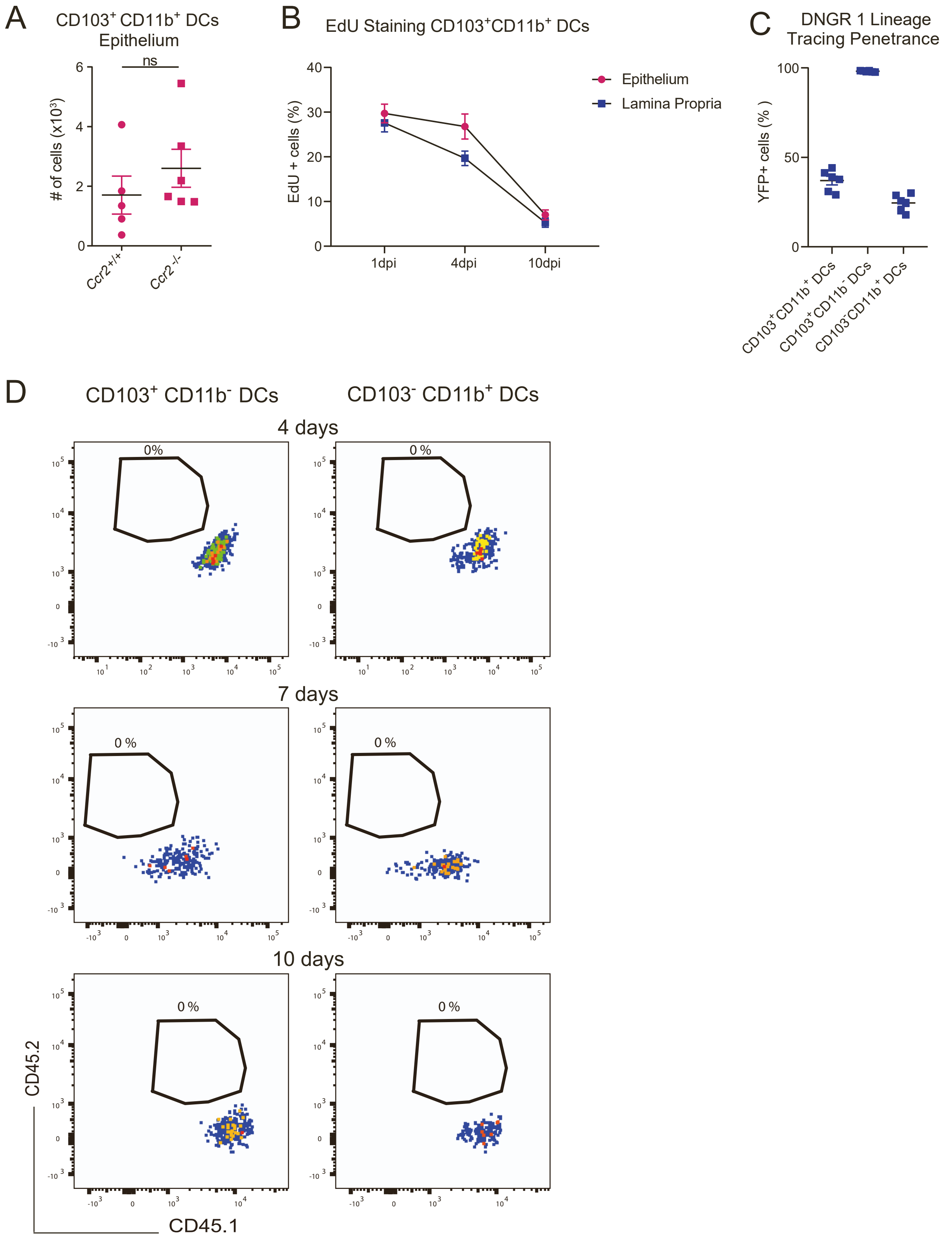


**Figure S1 (Related to Figure 3). Analysis of intraepithelial cDC ontogeny.** (**A**) Plots of number of CD103$^{+}$CD11b$^{+}$ in Live CD45$^{+}$ CD11c$^{+}$ MHCII$^{+}$ CD64$^{-}$ cells from the epithelium of the whole small intestine in *Ccr2*$^{+/+}$ and *Ccr2*$^{-/-}$ mice. Data are pooled from two independent experiments. Data were compared using t-test. Each symbol represents one mouse. (**B**) Intraepithelial and LP CD103$^{+}$CD11b$^{+}$ DCs lifespan analysis after a single EdU I.P. injection in C57BL/6J WT mice. Plot represent the percentage of EdU$^{+}$ cells after 1,4, and 10 days post injection. Date are pooled from 3 independent experiments with n = 2-3 mice per experiment. (**C**) Extent of DNGR 1 fate mapping measured as percentage of YFP$^{+}$ cells in CD103$^{+}$CD11b$^{+}$, CD103$^{+}$CD11b$^{-}$ and CD103$^{-}$CD11b$^{+}$cells (Live CD45$^{+}$ CD11c$^{+}$ MHCII$^{+}$ CD64$^{-}$) from the lamina propria of the whole small intestine of *Clec9a*$^{+/cre}$Rosa$^{+/EYFP}$ mice. (**D**) Representative flow cytometry dot plots of CD45.2 preDC transfer experiments showing differentiation dynamics in the small intestine epithelium of syngeneic CD45.1 mice. Differentiation of preDCs into CD103$^{+}$CD11b$^{-}$ and CD103$^{-}$CD11b$^{+}$ DCs was followed after 4, 7, and 10 days post-transfer.

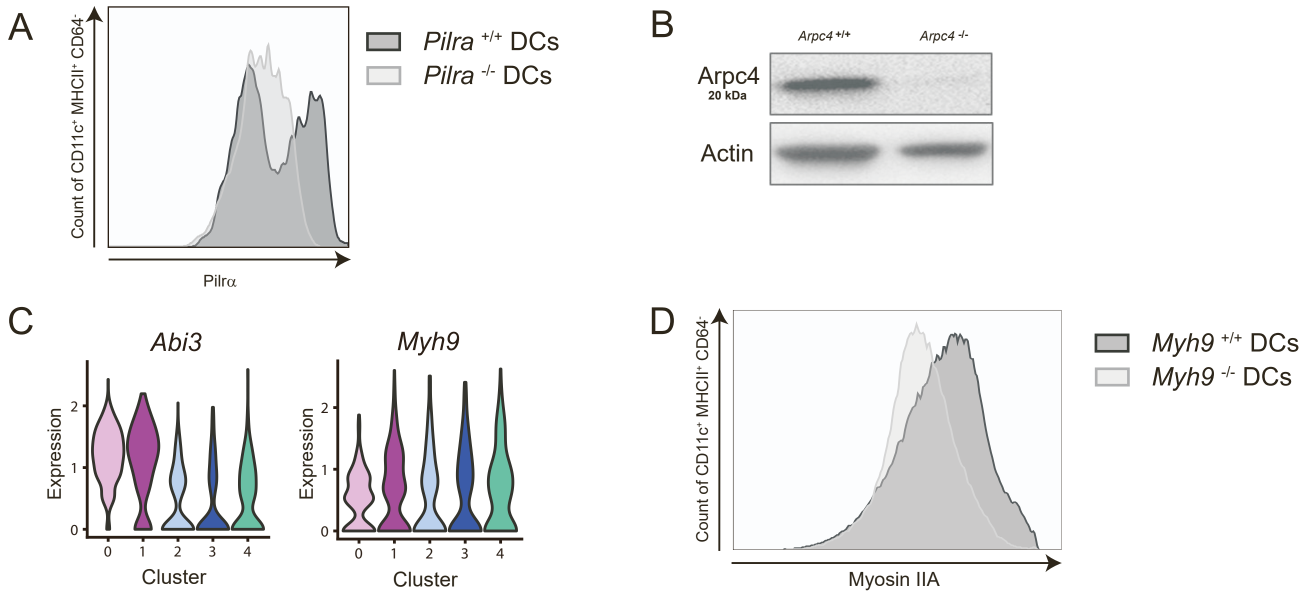


**Figure S2 (Related to Figure 4). Transmigration-related molecules characterization.** (**A**) Example of Pilralpha surface staining in $CD11c^{+}MHCII^{+}CD64^{-}$ cells from the small intestine of *Pilra*$^{+/+}$ and *Pilra*$^{-/-}$ mice. (**B**) Western blot of the Arp2/3 complex molecule Arpc4 show that *Arpc4*$^{flox/flox}$ x *Itgax*$^{Cre+}$ (*Arpc4*$^{-/-}$) cells do not express the Arpc4 protein in BM derived dendritic cells compared to the *Arpc4*$^{flox/flox}$ x *Itgax*$^{Cre-}$ (*Arpc4*$^{+/+}$) counterpart. (**C**) Violin plots representing expression of *Abi3* and *Myh9* (MyoIIA) among clusters. (**D**) Example of intracellular Myosin IIA staining in $CD11c^{+}MHCII^{+}CD64^{-}$ cells from the small intestine of *Myh9*$^{+/+}$ (*Myh9*$^{flox/flox}$ x *Itgax*$^{Cre-}$) and *Myh9*$^{-/-}$ (*Myh9*$^{flox/flox}$ x *Itgax*$^{Cre+}$) mice.

Figure S3

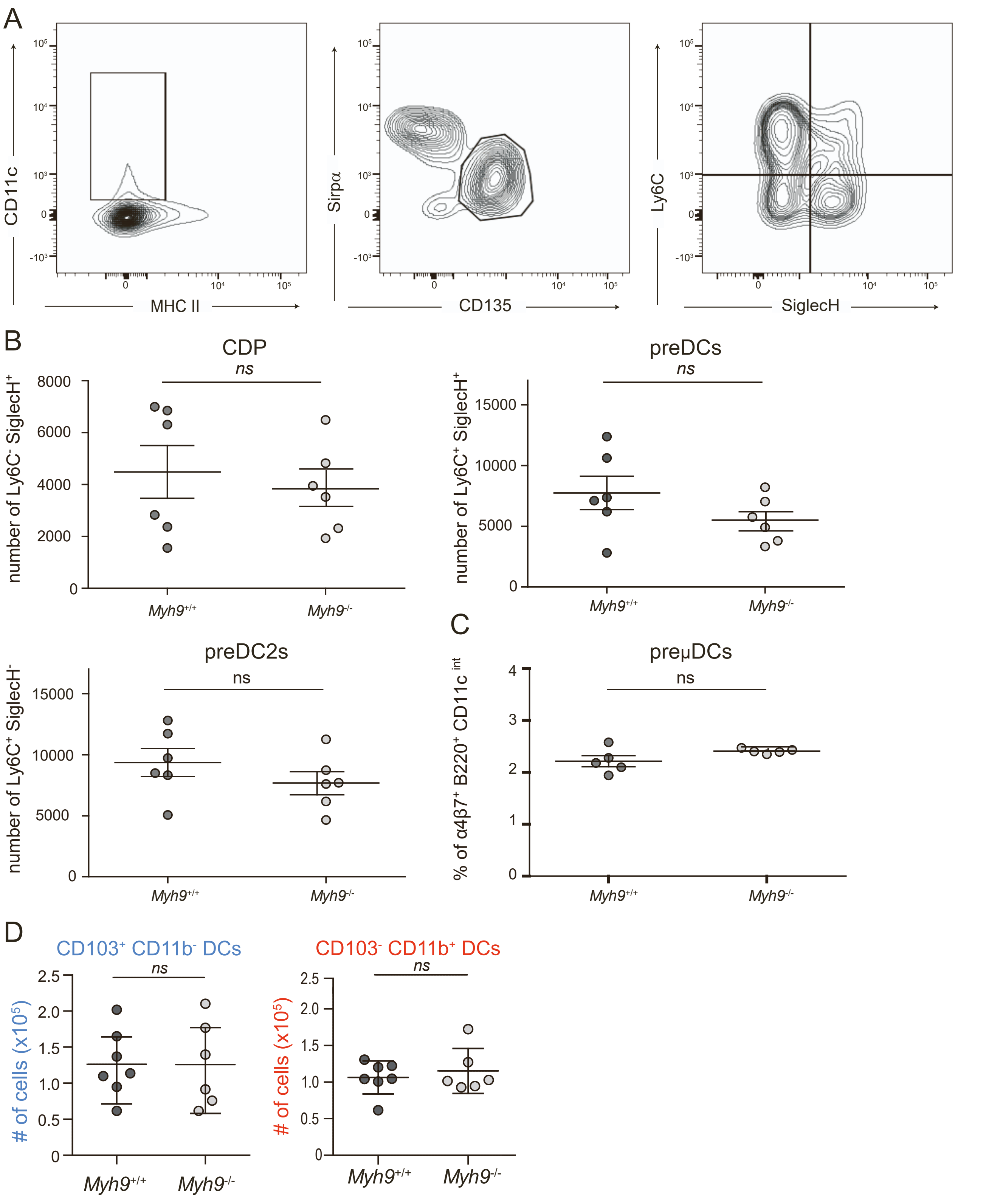


**Figure S3 (Related to Figure 4). *$Myh9^{-/-}$* mice characterization.** (**A**) Gating strategy used to characterize common DC progenitors (CDP) and preDCs from Live Lineage (CD3, CD19, NKP46, Ter119, Ly6G, B220) negative cells in bone marrow of *$Myh9^{+/+}$* (*$Myh9^{flox/flox}$* x *$Itgax^{Cre-}$*) and *$Myh9^{-/-}$* (*$Myh9^{flox/flox}$* x *$Itgax^{Cre+}$*) mice. (**B**) Plot of number of $Ly6C^-SiglecH^+$ in Live $Lineage^-$ cells (CDP) (left top panel), $Ly6C^+SiglecH^+$ in Live $Lineage^-$ cells (preDCs) and $Ly6C^+SiglecH^-$ in Live $Lineage^-$ cells (preDC2s), in bone marrow of *$Myh9^{+/+}$* and *$Myh9^{-/-}$* mice. Data are pooled from two independent experiments and compared using Paired t-test and Welch's test. Each symbol represents one mouse. (**C**) Plot of percentage of $\alpha4\beta7^+B220^+CD11c^{int}$ in Live Lineage (CD3, CD19, NKP46, Ter119, Ly6G) negative cells (preµDCs) in bone marrow of *$Myh9^{+/+}$* and *$Myh9^{-/-}$* mice. Data are pooled from two independent experiments and compared using Paired t test. Each symbol represents one mouse. (**D**) Plots of number of $CD103^+CD11b^-$ (cDC1s) and $CD103^-CD11b^+$ (classical cDC2s) in Live $CD45^+$ $CD11c^+$ $MHCII^+$ $CD64^-$ cells from the lamina propria of the whole small intestine in *$Myh9^{+/+}$* and *$Myh9^{-/-}$* mice. Data are pooled from three independent experiments. Data were compared using t-test. Each symbol represents one mouse.

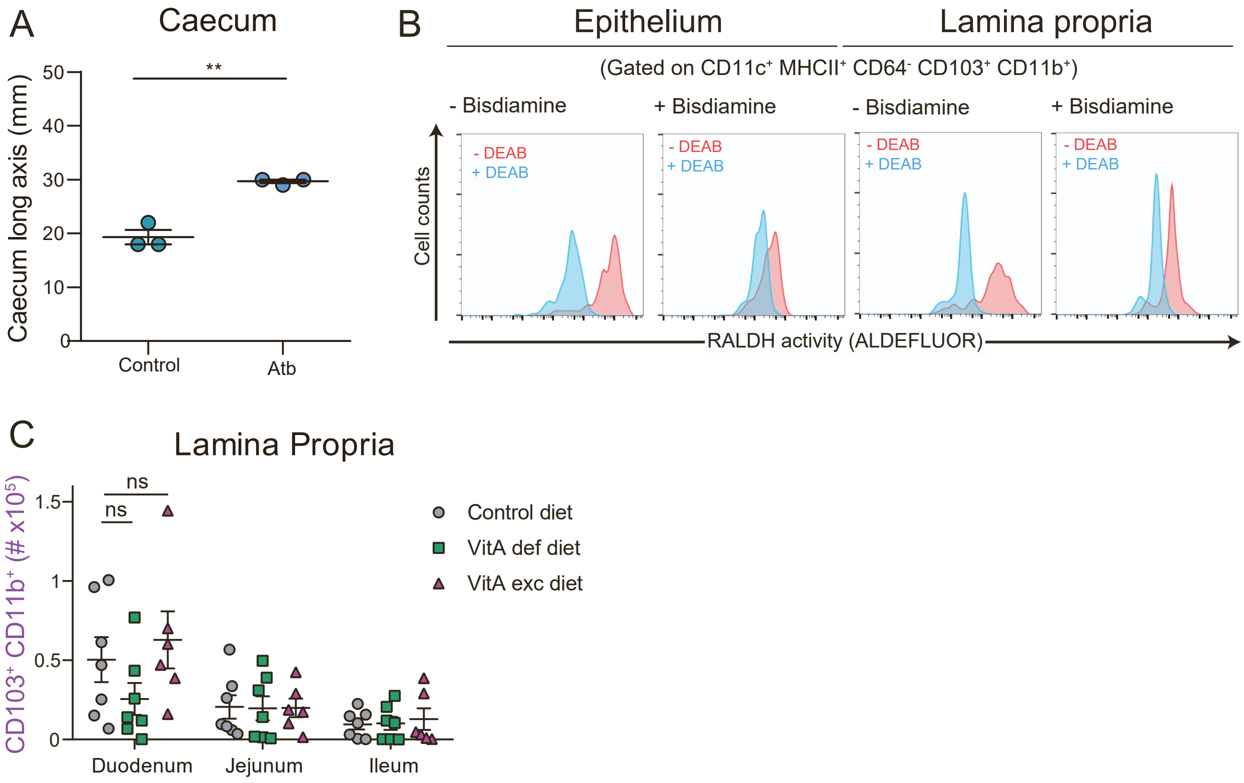


**Figure S4 (Related to Figure 5). Environmental factors modulation of cDC2 transmigration.** (**A**) Caecum size of adult SPF C57BL/6J mice gavage with PBS (control) or a cocktail of antibiotics. Data were compared using t-test. (**B**) Plots represent an example of epithelium (left) and lamina propria (right) from the small intestine of an adult SPF C57BL/6J mice treated with Bisdiamine or olive oil only. RALDH inhibitor (DEAB) provided with ALDEFLUOR kit was used as a negative control to set up gates for each sample. (**C**) Flow cytometry analysis of CD103$^{+}$CD11b$^{+}$ DCs numbers from the small intestinal lamina propria analyzed in duodenum, jejunum and ileum of SPF C57BL/6J mice fed with Vitamin A deficient, excess or control diet for 3 months. Data are pooled from 2 independent experiments and compared using two-way ANOVA. Each symbol represents one mouse.

Figure S5

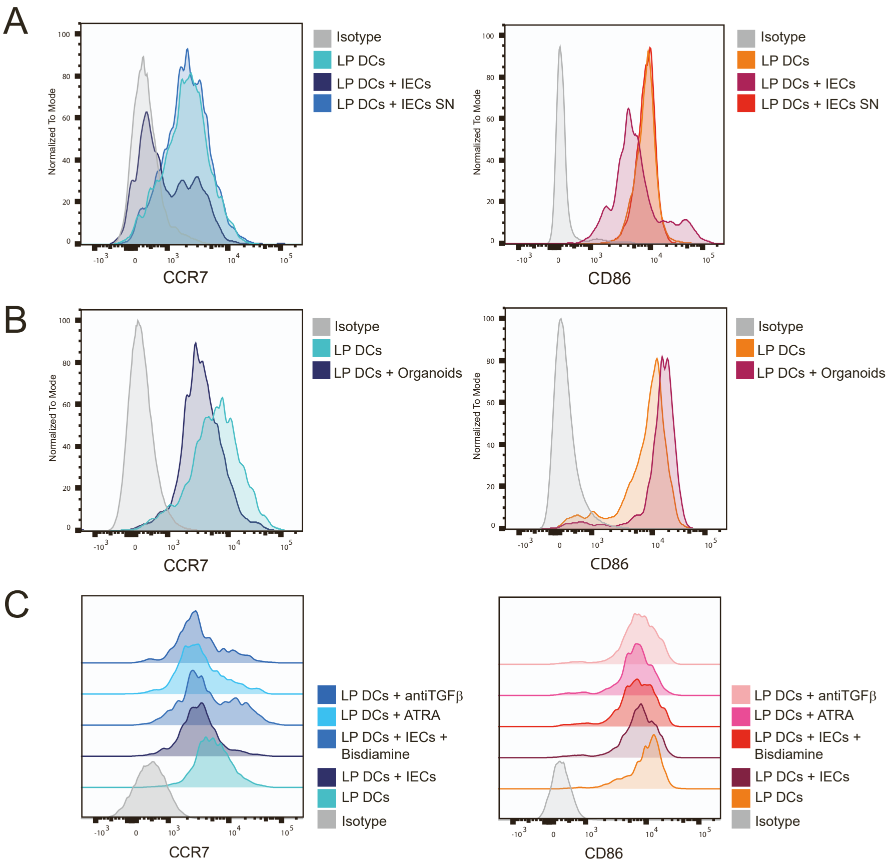


**Figure S5 (Related to Figure 6). CD103$^{+}$CD11b$^{+}$ cDC2s epithelial imprinting.** (**A**) Histogram plots representing an example of CCR7 and CD86 expression after co-culture of LP CD103$^{+}$CD11b$^{+}$ DCs with small intestine epithelial cells or supernatant of small intestine epithelial cells. (**B**) Histogram plots representing an example of CCR7 and CD86 expression after co-culture of LP CD103$^{+}$CD11b$^{+}$ DCs with small intestinal duodenal organoids. (**C**) Histogram plots showing examples of CCR7 and CD86 expression after incubation of LP CD103$^{+}$CD11b$^{+}$ DCs with small intestine epithelial cells in the presence or absence of ATRA, Bisdiamine and anti TGFβ treatments.

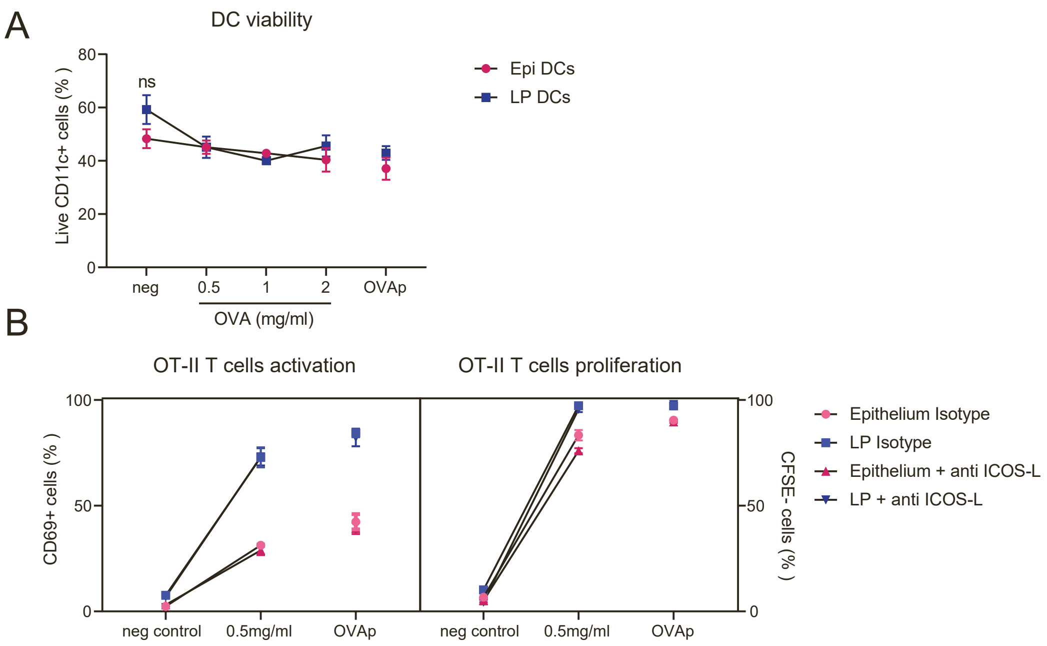


**Figure S6 (Related to Figure 7). Blocking ICOS-L does not increase T cell activation or proliferation induced by intraepithelial cDC2s.** (**A**) Percentage of Live CD11c$^+$ cells in antigen presentation assays after overnight co-culture of CD103$^+$CD11b$^+$ DCs with OT-II T cells. Data are pooled from 2 independent experiments. (**B**) OT-II antigen presentation assay performed with sorted CD103$^+$CD11b$^+$ DCs from both small intestine lamina propria and epithelium from C57BL/6J mice, showing percentage of activation (Left panel) and proliferation (Right panel) after incubation of DCs with anti ICOS-L and isotype antibodies at the same concentration (50 ug/ml). Data are pooled from 2 independent experiments.
